# The Chaperonin GroEL: A Versatile Tool for Applied Biotechnology Platforms

**DOI:** 10.3389/fmolb.2018.00046

**Published:** 2018-05-15

**Authors:** Pierce T. O'Neil, Alexandra J. Machen, Benjamin C. Deatherage, Caleb Trecazzi, Alexander Tischer, Venkata R. Machha, Matthew T. Auton, Michael R. Baldwin, Tommi A. White, Mark T. Fisher

**Affiliations:** ^1^Department of Biochemistry and Molecular Biology, University of Kansas Medical Center, Kansas City, KS, United States; ^2^Division of Hematology, Department of Internal Medicine, Mayo Clinic, Rochester, MN, United States; ^3^Department of Molecular Microbiology and Immunology, University of Missouri, Columbia, MO, United States; ^4^Department of Biochemistry, University of Missouri, Columbia, MO, United States; ^5^Electron Microscopy Core Facility, University of Missouri, Columbia, MO, United States

**Keywords:** chaperonin GroEL, electron microscopy, tilt series, tetanus neurotoxin, anthrax toxin, von Willebrand Factor, biolayer interferometry, cryoSPARC

## Abstract

The nucleotide-free chaperonin GroEL is capable of capturing transient unfolded or partially unfolded states that flicker in and out of existence due to large-scale protein dynamic vibrational modes. In this work, three short vignettes are presented to highlight our continuing advances in the application of GroEL biosensor biolayer interferometry (BLI) technologies and includes expanded uses of GroEL as a molecular scaffold for electron microscopy determination. The first example presents an extension of the ability to detect dynamic pre-aggregate transients in therapeutic protein solutions where the assessment of the kinetic stability of any folded protein or, as shown herein, quantitative detection of mutant-type protein when mixed with wild-type native counterparts. Secondly, using a BLI denaturation pulse assay with GroEL, the comparison of kinetically controlled denaturation isotherms of various von Willebrand factor (vWF) triple A domain mutant-types is shown. These mutant-types are single point mutations that locally disorder the A1 platelet binding domain resulting in one gain of function and one loss of function phenotype. Clear, separate, and reproducible kinetic deviations in the mutant-type isotherms exist when compared with the wild-type curve. Finally, expanding on previous electron microscopy (EM) advances using GroEL as both a protein scaffold surface and a release platform, examples are presented where GroEL-protein complexes can be imaged using electron microscopy tilt series and the low-resolution structures of aggregation-prone proteins that have interacted with GroEL. The ability of GroEL to bind hydrophobic regions and transient partially folded states allows one to employ this unique molecular chaperone both as a versatile structural scaffold and as a sensor of a protein's folded states.

## Introduction

The complete functional GroE chaperonin system (GroEL, GroES) is an exquisite allosteric machine that can initially capture transient hydrophobic pockets on folded proteins or partially unfolding protein intermediates. If the size of the captured protein is sufficiently small (<50 kDa), the folding intermediates are released into the highly-structured GroEL-GroES (GroE) nanochamber where folding can continue (Gruber and Horovitz, 2016). Although initial structural work clearly indicates that the interior of the GroEL nanochamber becomes more hydrophilic to aid the folding reaction (Saibil et al., 2013), there is new evidence suggesting that this simple explanation needs to be amended. Specifically, there is increasing mechanistic and structural evidence indicating the unstructured hydrophobic Gly-Gly-Met tetra-repeat C-terminal tails play an important kinetic/structural role in both binding and unfolding the captured protein (Weaver et al., 2017). ATP nucleotide hydrolysis results in timed allosteric disruption of the GroE nanochamber to release the protein substrate back into solution. In the absence of any nucleotide (ATP or ADP), GroEL is capable of both capturing and arresting the folding of any protein that is either completely unfolded or, more interestingly, fluctuating in a dynamic equilibrium between folded and partially folded states (Viitanen et al., 1991; Smith and Fisher, 1995; Smith et al., 1998). As shown initially by Martin and Hartl and later by the Valpuesta group, the physiological relevant capture of transient dynamic states could be particularly relevant for organismal survival to prevent mass aggregation during heat stress (Martin et al., 1992; Llorca et al., 1998).

The nucleotide-free form of GroEL is one of the most promiscuous binders of partially folded proteins that has been encountered to date. Indeed, early purification schemes for GroEL were plagued by co-purification of large and diverse amounts of contaminating proteins and peptides. These contaminants were bound tightly to the now nucleotide-free GroEL as endogenous ATP was diluted and depleted during cell disruption. Early visual analysis by the Lorimer group of the GroEL-captured proteins revealed the contaminating proteins were nascent, unfolded *E. coli* proteins (Viitanen et al., 1992). Clark and Frieden further analyzed these contaminants using mass spectroscopy and found a substantial amount of short, cleaved polypeptides were also present prior to removal during final purification steps (Clark et al., 1998). Using a very clever *in vivo* experimental design, the Horwich group generated a temperature sensitive GroEL mutant which immediately loses its ability to adopt its nucleotide-bound low affinity state upon upshift to non-permissive temperatures (Sewell et al., 2004). Using this mutant, it was found that a significantly large number of nascent *E. coli* proteins (estimated >330 proteins by MudPIT analysis) aggregated or co-precipitated with GroEL *in vivo* (Chapman et al., 2006). Curiously, EM analysis of *E. coli* containing this temperature-sensitive mutant showed highly structured, regular arrays within the cytoplasm. These arrays look quite similar to the inclusions found in some mitochondrial diseases, such as ragged red fiber syndrome, where protein homeostasis and production of the electron transport chain proteins are disrupted. Independently, our laboratory confirmed that large protein substrates with multiple opposing hydrophobic surfaces can induce chaining into extended linear arrays when captured by GroEL and these chains are easily visualized by negative-stain EM (Akkaladevi et al., 2015). The key finding from all these experimental observations demonstrates that the nucleotide-free GroEL can capture a wide variety of partially folded proteins with high affinity.

The promiscuous nature of the nucleotide-free chaperonin has been used as a tool to capture and isolate transient protein folding intermediates with the intent to prevent aggregation during concentration. This capture and pause in folding permits folding of target proteins at high concentrations (Fisher, 1993). Notably, capture and successful folding using the chaperonin was accomplished in the mg/mL range as compared to the limited success of only observing folding at the μg/mL range in the absence of the chaperonin (Fisher, 1993; Smith and Fisher, 1995). The chaperonin can be rapidly separated from the substrate protein following ATP release either by attaching GroEL to beads and spinning down (Voziyan et al., 2005) or by immunoprecipitating GroEL (Fisher and Yuan, 1994; Fisher, 1998).

The presence of the nucleotide-free chaperonin can be used to prevent protein aggregation resulting from the presence of dynamic transient states. This aggregation is dependent on both the lifetime of the transient state and the interaction between one of these transient open hydrophobic states with other transient open states. The population of open states results from naturally occurring native structural fluctuations that persist longer in missense mutation disease states. These open, aggregation prone states can collide with one another to form initial small aggregates (dimers or greater) that can either be reversible or irreversible depending on the strength of the protein-protein interaction (Roberts, 2014). This aggregation process is particularly problematic for biotherapeutic proteins where the requirement of a long shelf life provides ample opportunities for aggregation to occur. In relation to human health, if similar deleterious long-lived transient misfolding processes occurs within cells, this can lead to detrimental protein aggregation or abnormal protein clearance *in vivo*, ultimately resulting in a protein folding disease phenotype.

In this work, additional experiments are presented that extend our use of GroEL biosensor technologies to (1) detect partially folded populations of mutant-type when mixed with wild-type counterparts, (2) detect differences between wild-type, gain of function, and loss of function folding disease mutations and, (3) utilize GroEL molecular scaffolds to capture or maintain solubility of aggregation prone proteins for EM structural analysis. Specifically, in the first example, GroEL biosensors detect and can potentially quantitate the amount of partially folded mutant-type maltose-binding protein (MBP) when mixed with wild-type. Furthermore, the data demonstrate that this detection response is linear with respect to the amount of mutant-type protein within a low concentration range. The second application expands on our previous work comparing wild- and mutant-type proteins. Using the automated denaturant pulse protocol, one can rapidly assess differences through the acquisition of distinct and separate kinetically controlled denaturation isotherms. In the case presented herein, this comparison is made between wild-type and two missense folding disease mutants for von Willebrand factor (vWF). In the final application, GroEL is used as a scaffold to aid negative-stain EM and tilt series image acquisition to construct low-resolution structures of the aggregation-prone tetanus neurotoxin (TeNT) from a mixed population while also visualizing the GroEL-TeNT complexes.

## Materials and methods

### Materials

Bovine Serum Albumin Fraction V (BSA FV) (Sigma Aldrich A9647) was diluted to 1 mg/mL with GroEL Buffer (GB) from a 300 mg/mL BSA FV stock in ultrapure (18.2 MΩ) water. All other materials (buffer agents and salts) were obtained from Fisher Scientific. The cryoSPARC system (Structura Biotechnology) is run on a single Supermicro 4U workstation equipped with 2x NVIDIA Titan Xp GPUs, Intel® Xeon® E5-1630 v4 processor, 4x 16 GB DDR4-2400 RAM, 1.2 TB Intel® SSD DC S3610 for runtime caching, and 4x 4 TB Seagate HDD for data storage (Silicon Mechanics assembled), which is housed in lab.

### GroEL purification

GroEL was purified following protocols outlined previously (Voziyan and Fisher, 2000; Lea et al., 2016). GroEL stock solutions were stored in GroEL buffer (50 mM Tris, 50 mM KCl, 10 mM MgCl_2_, 0.5 mM EDTA, pH 7.5) at 50 μM tetradecamer with 50% glycerol at 4°C.

### MBP purification

Wild-type and W169G MBP were expressed in *E. coli* and purified using methods described previously for His_6_ tagged proteins (Xia et al., 2013). Purified protein was stored in 20 mM phosphate buffer at pH 7.0 with 100 mM NaCl.

### vWF A1-A2-A3 purification

Wild- and mutant-type vWF A1-A2-A3 tridomains with von Willebrand Disease point mutations (V1314D and F1369) engineered into the A1 domain (Tischer et al., 2014) were expressed with a C-terminal His_6_ tag on the A3 domain and purified as previously described (Auton et al., 2007a). Purified protein was stored in vWF buffer (25 mM TRIS, 150 mM NaCl, pH 7.5) at 4°C and used within 2 weeks.

### TeNT purification

Tetanus neurotoxin was purified in the Baldwin laboratory as previously described (Burns and Baldwin, 2014). In brief, site directed mutagenesis was performed to remove the catalytic residues (R372A/Y375F). TeNT(RY) was expressed in *E. coli* and cell lysate was passed over both Ni-NTA and Strep-Tactin resins to twin affinity purify the neurotoxin. Purified protein was concentrated to 1.5 mg/mL in TeNT buffer (30 mM HEPES, 500 mM NaCl, pH 7.6) and frozen at −80°C until use.

### GroEL-BLI biosensor construction

Preparation of the GroEL-BLI biosensor followed similarly to methods previously described (Naik et al., 2014; Pace et al., 2018). GroEL stock solution was buffer exchanged with GroEL buffer (GB) using an Amicon Ultra-4 30 MWCO to remove glycerol and monomers. The exchanged GroEL was then biotinylated using NHS-PEG12-Biotin (Thermo Scientific 21312) at a 20:1 biotin:GroEL ratio for 30 min at room temperature. Biotinylated GroEL (bGroEL) was then buffer exchanged into fresh GB in order to remove excess biotinylation reagent from solution. The GroEL-BLI biosensor was prepared in the beginning of each BLI run by hydrating a streptavidin tip (*forté*Bio 18-5019) in GB for 10 min, followed by a 1 min binding step in 0.5 μM bGroEL with a wash step in GB for 30 s to remove non-bound bGroEL. Loading amplitudes were 5–6 nm indicating saturation of the biosensor surface. As a precaution, GroEL tips were incubated in a BSA solution to block non-specific binding sites (Naik et al., 2014; Pace et al., 2018). Natively folded BSA does not bind to GroEL or inhibit its ability to bind hydrophobic patches. The GroEL-BLI biosensor was then used for subsequent experiments.

### Standard curve construction for MBP experiments

The following seven steps were performed using the BLItz to generate a standard curve using varying MBP concentrations (0.5, 1.0, 1.5, 2.0 μM) for both wild-type and W169G:

**Table:** 

**Step**	**Time (s)**	**Event**	**Composition**
1	30	Initial Baseline	GroEL Buffer (GB)
2	300	Loading	0.5 μM bGroEL in (GB)
3	30	Baseline	GB
4	300	Custom	1 mg/mL BSA FV in GB
5	30	Baseline	GB
6	300	Association	MBP WT/W169G/Mix in GB
7	300	Dissociation	GB

For wild-type/W169G mixture, concentrations of 0.5, 1.0, 1.5, and 2.0 μM W169G MBP were tested in the presence of 0.5 μM wild-type MBP in order to determine whether the mixture would more closely resemble either the wild-type or W169G MBP response. Each sample concentration for the above experiments was tested three times for reproducibility. Experiments were all conducted at room temperature. Binding responses were extracted at 988.50 s into each run, or 298.50 s into the association step in the above table so as to diminish small contributions from buffer induced refractive index changes during the dip and read procedure.

### Z′ factor calculation

Z′ factor (read Z prime factor) is calculated for high throughput screening using the formula:

$$\begin{array}{l} Z^{\prime }Factor=\;1\;-\;\frac{3\left({\hat{\sigma }}_{1}+\;{\hat{\sigma }}_{2}\right)}{\left|{\hat{\mu }}_{1}-\;{\hat{\mu }}_{2}\right|} \end{array}$$

In the formula above, ${\hat{\sigma }}_{1}$, ${\hat{\sigma }}_{2}$, ${\hat{\mu }}_{1}$, and ${\hat{\mu }}_{2}$ are the sample standard deviations and sample means, respectively, for condition 1 and condition 2. In this study, binding response is the input with condition 1 being pure wild-type and condition 2 being pure mutant-type population. Z′ factors can range from 0 to 1 with any score of 0.5 or better indicating those conditions are appropriate for assay development. If the sample standard deviations are equal, a score of 0.5 indicates there are 12 standard deviations separating the sample means. This data was collected on the single-channel BLItz unit and the assay sensitivity and reproducibility can be improved if the eight-channel Octet is utilized (Lea et al., 2016).

### Denaturant pulse assay for vWFA1-A2-A3

**Figure 1:**
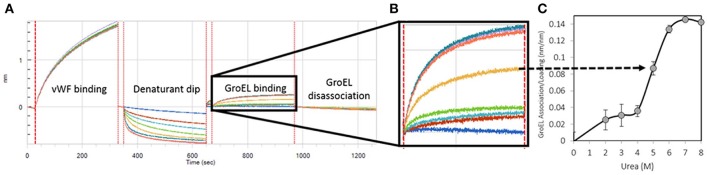
Generation of a Kinetically Controlled Denaturant Pulse Isotherm for the Wild-type von Willebrand Factor A1-A2-A3 Triple Domain Fragment. **(A)** Protein is partially denatured during automated urea pulse steps, and **(B)** the GroEL binding amplitude was **(C)** plotted as a function of urea concentration to generate a kinetically controlled denaturation isotherm (Lea et al., 2016).

A sample complete run for von Willebrand Factor is represented in Figure 1A. This triple A domain protein (A1-A2-A3-His_6_ tag) was attached to a Ni-NTA BLI biosensor in an orientation where the A3 domain is closest to the biosensor surface. The vWF denaturant pulse assays were performed on an automated eight-channel Octet RED96 instrument (*forté*BIO) shaking at 1,000 rpm, 25°C. For detailed programming of the denaturant pulse system, see our previous publication (Lea et al., 2016). The programmed steps were as follows with the urea range from 0 to 7 M by 1 M step:

**Table:** 

**Step**	**Time (s)**	**Event**	**Composition**
1	30	Initial Baseline	vWF Buffer (vWFB)
2	300	Loading	0.6 μM vWF Protein in vWFB
3	30	Baseline	vWFB
4	600	Custom	Urea Range
5	10	Baseline	GroEL Buffer (GB)
6	300	Association	0.5 μM GroEL in GB
7	300	Dissociation	GB
8	5	Regeneration	10 mM glycine, pH 1.7
9	5	Regeneration	GB
10	5	Regeneration	10 mM glycine, pH 1.7
11	5	Regeneration	GB
12	5	Regeneration	10 mM glycine, pH 1.7
13	5	Regeneration	GB
14	60	Regeneration	10 mM NiCl_2_

Runs were performed in triplicate with tip regeneration performed after runs 1 and 2 only. The GroEL binding signal was plotted as a function of denaturant concentration to create kinetically controlled denaturation isotherms for wild-type, gain of function (V1314D), and loss of function (F1369I) mutant vWF (Figures 1B,C). For Figure 1, Step 4 above was run for 300 s resulting in less GroEL association as compared with the GroEL binding amplitudes presented in **Figure 4**. The denaturant pulse isotherms for both experiments followed the same trend (data available upon request).

### Transmission electron microscope sample preparation

Two hundred mesh carbon-coated copper grids (Electron Microscopy Sciences CF200-Cu) were glow discharged for 20 s at −15 mA in 39 mBar atmosphere. After glow discharge, grids were rested for approximately 20 min before sample application. 4 μL of sample was applied to the rested grid for 1 min and then wicked off using Fisherbrand™ P8 Grade filter paper. Grids were washed once with ultrapure water and wicked off as fast as possible. The grids were then stained for 5 s with 0.022 μm filtered 0.75% uranyl formate (Electron Microscopy Sciences 22451) in ultrapure water and then wicked dry. Grids were completely dried overnight on filter paper inside a 100 mm Petri plate. All EM images were acquired using 100 keV JEOL-JEM 1400 transmission electron microscope aside from the tilt series.

### Tilt series acquisition and alignment

A pre-made grid was loaded into a single tilt axis holder and inserted into a FEI Tecnai F30 G2 Twin transmission electron microscope. After beam alignment, the grids were manually scanned for well-isolated complexes. Once found, a tilt series was taken from 0° to +60° and then 0° to −60° every 2°. The series was combined in order by the microscope's capture software. The single .mrc stack file was separated into single micrograph .mrc files using *excludeviews* command from IMOD (Kremer et al., 1996). The single frames were manually realigned using midas from IMOD. Frames −60° to −56° and +58° to +60° were excluded from final processing as the grid bar blocked the electron beam.

### GroEL-TeNT ATP release sample preparation

The following steps were run on the single channel BLItz unit:

**Table:** 

**Step**	**Time (s)**	**Event**	**Composition**
1	30	Initial Baseline	GroEL Buffer (GB)
2	60	Loading	0.5 μM bGroEL in GB
3	30	Baseline	GB
4	600	Association	0.5 μM TeNT in GB
5	30	Baseline	GB

After the run was completed, the biosensor tip was transferred into a PCR tube containing GB + 450 mM NaCl + 10 mM ATP (stock solution at pH 7.5) for 10 min to release the captured TeNT. After release, the sample was stained for EM without resting the grid first and with 2 additional wash steps between sample and stain to remove phosphate ions, which precipitates uranyl ions.

### GroEL-mAb complex sample preparation

Modified biotin GroEL biosensors were prepared exactly as it would be for normal GroEL biosensors with the sole difference the replacement of NHS-PEG12-Biotin with NHS-SS-Biotin. Once adequate binding of the IgG was observed via BLI, the tip was manually held in a 3 μL drop of 50 mM DTT for 20 s. The sample was then processed as normal for EM analysis.

### GroEL-TeNT thermal sample preparation

Equimolar concentration (500 nM) of TeNT and GroEL were mixed in GroEL Buffer and incubated at 25°C 200 rpm for 24 h. Samples were diluted to 7 nM and stained as described.

### TeNT reconstruction using cryosparc system

Single particle reconstructions were performed on images taken on a 100 keV JEOL-JEM 1400 transmission electron microscope of thermal GroEL-TeNT complex grids. EMAN2 was used to pick particles, perform CTF correction, generate structure factor, and generate .star files for future processing (Tang et al., 2007). The data from 537 particles was then imported into the cryoSPARC system using the .mrc and .star files (Punjani et al., 2017). *Ab initio* modeling and one round of refinement were performed in cryoSPARC using default settings aside from double the pre- and post-annealing default iterations. Twenty-two angstrom (22 Å) resolution was generated using a 0.143 FCS score. cryoSPARC was used for the tilt series reconstruction using the same steps, but the input images were of only 28 TeNT particles, with each particle having 57 views, corresponding to each angle in the tilt series. These views from the tilt series were input as individual 3D particles. The total number of 2D tilted views that went in the reconstruction of the tilt series generated dataset was 1,500. The I-TASSER structure (Zhang, 2008) was fit into both the single particle reconstruction and tilt series electron density maps using molecular dynamics flexible fitting (Trabuco et al., 2008).

## Developing GroEL into a direct biosensor to detect partially folded protein populations within protein mixtures

### GroEL biosensors

Noting the promiscuous capture efficiency of the chaperonin GroEL, biosensors were developed with the goal of detecting protein populations that possess exposed hydrophobic regions. Many proteins exist as dynamic equilibrium mixtures of folded and hydrophobic, partially folded species. These hydrophobic entities (and regions) can either exist as long-lived, stable species or flickering, transient species that appear and disappear as a function of time. It is possible to detect the presence of transiently unfolded species that exist for a long enough window of time to collide with GroEL in their open state. The existence of this fluctuating equilibrium is particularly important when evaluating the integrity of protein populations that may be susceptible to slow aggregation reactions. Protein populations that expose transient hydrophobic patches are often the primary event that eventually leads to deleterious aggregation. This makes this approach relevant when determining if pre-aggregation hydrophobic species are present in concentrated biotherapeutic formulations. The binding site of GroEL can accommodate domains of large protein to detect partially folded hydrophobic regions which cannot fully enter the nanochamber. The visualization of the GroEL-protein complexes may be useful in identifying regions that lead to aggregation during long-term storage.

**Figure 2:**
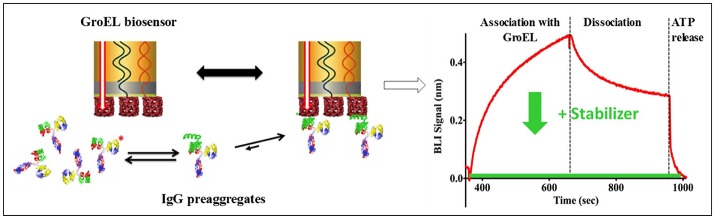
General Scheme on the Use of Biolayer Interferometry and GroEL Biosensor. The biosensor can be used to detect the appearance of pre-aggregates (here shown on the left as IgG localized unfolding (green helix). The GroEL biosensor can capture the unfolded region even though the majority of the IgG is properly folded. On the right-hand side, a representative sensogram is shown where association and dissociation phases can easily be seen. The dissociation is non-specific interactions. This is confirmed by specific interaction reversal by ATP addition. The green curve demonstrates how a specific stabilizer could limit IgG association with GroEL.

The pharmaceutical industry is intensely interested in stabilizing biotherapeutic proteins to increase shelf-life, enhance or engineer product stability, increase drug delivery efficacy, and to avoid patient immune response against the biotherapeutic protein arising from aggregation products. The development of the biolayer interferometry (BLI) GroEL biosensor permits direct assessment of protein stability in concentrated protein solutions of biotherapeutics or related examples (Naik et al., 2014; Pace et al., 2018). BLI label-free methods are preferable over surface plasmon resonance (SPR) methods because the former technique does not rely on microfluidic flow through microchannels. The latter method (SPR) can be easily compromised by aggregation events, resulting in microchannel blockage and fouling. In addition to the aggregation phenomenon, high protein concentrations will result in immense refractive index changes during an SPR run that must be subtracted from the sensogram. The scheme illustrated in Figure 2 demonstrates a typical BLI GroEL biosensor output sensogram. A unique feature of this GroEL biosensor system is the release of the partially folded protein or hydrophobic transients from the biosensor surface during ATP incubation. This ATP dependent reversal of protein binding to GroEL results in a return to the baseline before capture indicating that the GroEL-protein interaction specific for the GroEL binding site (Naik et al., 2014; Pace et al., 2018). If stabilization of the protein is achieved either through the alteration of the formulation solution, the addition of stabilizing ligand, or genetic engineering, the capture by the GroEL biosensor is diminished, sometimes quite dramatically (Naik et al., 2014; Lea et al., 2016; Pace et al., 2018).

**Figure 3:**
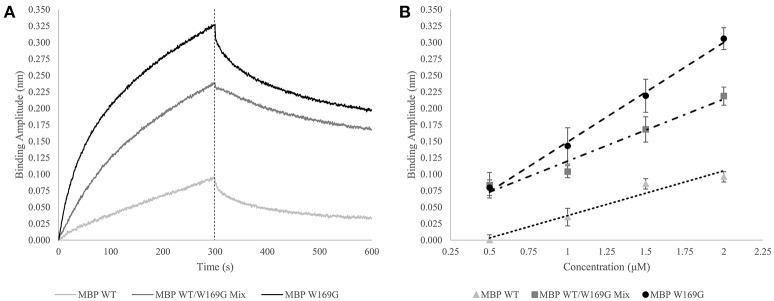
Wild- vs. Mutant-Type MBP Capture by GroEL Biosensor. **(A)** Raw biosensor traces at 2 μM for wild-type (light gray), W169G (black), and the mixed population (dark gray). The dotted line indicates the end of association, which is the amplitudes used for the linear response. **(B)** Linear response of wild-type and destabilized mutant-type MBP capture versus concentration (triangles and circles, respectively). For pure protein population, the x-axis is absolute concentration. Dose-dependent response of doping mutant-type MBP into wild-type is shown in squares. For the protein mixtures, the x-axis value is the concentration of mutant-type added to a solution containing 0.5 μM wild-type. R-squared values for the wild-type, W169G, and mixture are 0.964, 0.996, and 0.961, respectively. The Z' factor for wild-type versus mutant-type response at 2 μM is 0.649, which indicates the response of this system is reliable to develop an assay that can distinguish between mixtures and homogenous proteins.

In an expansion of the use of the GroEL biosensor, it is of interest to determine if one can detect increases in mutant-type population within a mixture of wild- and mutant-type protein. Proof of concept experiments are presented in this section for the model protein maltose-binding protein (MBP). MBP with the W169G missense mutation is a less thermally stable protein, resulting in a dynamic equilibrium between folded and partially folded conformers. First, demonstration of a linear binding response with respect to the protein concentration after a certain time (Figure 3A – dotted line) is generated for pure populations of either wild- or mutant-type protein and the amplitudes are plotted as a function of concentration. Next, it is of interest to determine how this linear response of the mutant-type protein behaves when MBP species are mixed in solution. Experimental setups such as these may help determine if the mutant protein misfolding is independent or dependent of the presence of the wild type protein. In the latter situation, this type of an assay may be helpful in assessing the ability of mutant-type proteins to induce misfolding of stable wild-type species. The potential interactions between wild- and mutant-type folds leading to an induction of misfolding can be applicable toward understanding the impact of protein misfolding in some disease states. This situation is particularly relevant for instances where one mutant allele results in dominant negative phenotypes as seen with many tumor suppressor p53 mutations.

### Detection of mutant population within a mixed solution of wild- and mutant-type MBP

The GroEL biosensor detected the greatest increase in binding amplitude for the mutant population alone as compared with the synonymous concentration of wild-type MBP (Figures 3A,B). The mixed population biosensor binding amplitudes showed a slight decline in sensitivity as compared to the pure populations. However, the mixture of wild type and mutant protein still show an increased binding response as compared to the wild-type protein alone (Figure 3B). Additionally, this increased response is linear with respect to the concentration of mutant-type subpopulation with the shallower slope indicating the lessened sensitivity. Light scattering measurements at 320 nm showed no increase suggesting that the presence of large aggregate species is not prevalent in any of the mixture samples, supporting the notion that the binding amplitude from the GroEL biosensor was due to the presence of pre-aggregate subpopulations. This lessened sensitivity is most likely due to reduced collisional frequency effects simply from the presence of native folded MBP. The calculated Z' factor between the pure wild- and mutant-type populations at 2 μM concentration is 0.649. Any Z' factor above 0.5 indicates that this concentration and conditions is useful for assay development (Zhang et al., 1999; Lea et al., 2016).

Thus, it is possible to detect a change in binding signal due to the increased presence of less stable hydrophobic species. Disparities in linear response between mutant-type alone and wild-/mutant-type mixture populations are predicted to be substantially altered (diminished) if the wild- and mutant-type proteins interact to bury the exposed hydrophobic faces.

## Comparison of kinetically controlled denaturation isotherms of wild- and mutant-type von willebrand factor using GroEL-BLI denaturant pulse assay

### Denaturant pulse assay

The kinetic stability of aggregation-prone proteins can be determined using a unique chaperonin dependent denaturant pulse assay. This technique assesses the stability of proteins immobilized on BLI biosensor surfaces after a time-controlled pulse in various denaturant solutions. GroEL binding to hydrophobic patches on the unfolded protein amplifies the unfolded protein signal. The development of this method is discussed in a previous publication from this laboratory (Lea et al., 2016). Kinetically controlled denaturation isotherms were rapidly and reproducibly generated for several protein systems. In this current work, the denaturant pulse approach is expanded to encompass new and more complex systems. To illustrate the expanding utility of this method, denaturation isotherms of wild- and mutant-type von Willebrand Factor (vWF) triple A domain were collected and compared. Many missense mutants have a tendency to aggregate in solution during conventional stability analysis. Therefore, the immobilization of missense mutants prior to performing the denaturant pulse assay avoids this common difficulty.

### von willebrand factor

vWF is a multimeric plasma glycoprotein which initiates platelet adhesion at sites of vascular injury. Under high shear stress, vWF unravels and binds to platelets and collagen to create a plug to stop bleeding. von Willebrand Disease (vWD) is a bleeding disorder affecting approximately 1% of the world population. This hereditary disease is caused by mutations that cause quantitative deficiencies of vWF or qualitatively alter vWF function. Mutations in A1 of the triple A domain of vWF alter its specificity for the platelet receptor GP1bα. Mutations in A3 can affect its collagen binding affinity. Finally, mutations in A2 cause defective intracellular transport or enhance proteolysis of a scissile bond recognized by the soluble blood metalloprotease (ADAMTS13), which helps regulate the multimeric size of vWF (Keeney and Cumming, 2001). Some vWD mutations that change A1-GP1bα binding specificity result in local misfolding of the A1 domain (Tischer et al., 2014, 2017; Zimmermann et al., 2015; Machha et al., 2017) causing both gain and loss of function phenotypes. Two such mutations are V1314D, a gain of function mutation that causes increased platelet adhesion, and F1369I, a loss of function mutation that does not adhere to platelets at all. The GroEL-based BLI denaturant pulse assay was used to assess the kinetic stability of vWF A1-A2-A3 for both wild-type and partially disordered vWD point mutants.

### vWF wild- & mutant-type denaturant isotherms

**Figure 4:**
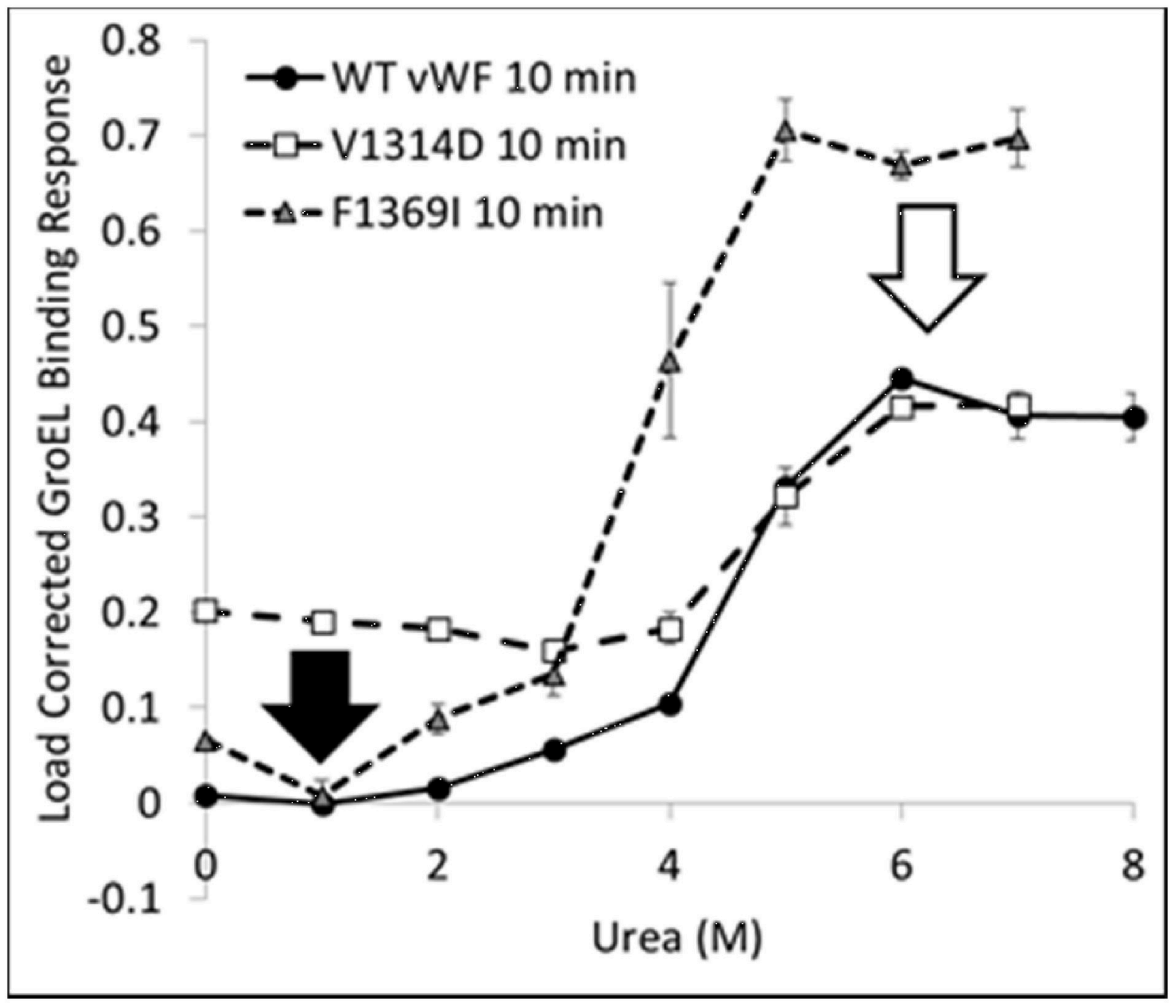
vWF Kinetically Controlled Denaturant Pulse Isotherms for Wild- and Mutant-Types. Kinetic denaturation isotherms for the wild-type (circles), gain of function mutant (squares), and loss of function mutant (triangle) overlaid for comparison. The closed and open arrows denote the potential corrective ligand induced responses that may restore wild-type denaturant pulse profile and protein function.

The kinetically controlled denaturant isotherms for wild-type, F1369I, and V1314D vWF triple A domains are shown in Figure 4. GroEL binding to these misfolded triple A domain variants was assessed as a function of urea concentration using a BLI denaturation pulse assay. vWF-GroEL association was confirmed with direct observation using EM (see section Release and EM Analysis of Proteins Released From Biosensor Surfaces). Although, the gain of function mutant V1314D had exposed hydrophobic patches under little or no denaturant as evidenced by GroEL association at these urea concentrations, the denaturant pulse profiles for wild-type and V1314D were similar at high urea concentrations. By contrast, F1369I and wild-type had similar GroEL binding at low urea concentrations, but at high denaturant conditions the F1369I exhibited significantly higher binding by GroEL.

In equilibrium denaturation profiles using urea, the thermodynamics of vWF triple A domain unfolding has been described as three domains linked in a linear fashion in which unfolding of each domain proceeds orderly with the simultaneous unfolding of A1 and A2 at low urea followed by A3 at high urea (Auton et al., 2007b). Evidence suggests that in the absence of urea, the A1, A2, and A3 domains interact with each other and mutations can disrupt these interactions as a result of their intrinsic effects on the single domain (Auton et al., 2010). It is possible that the interactions between domains observed in the wild-type protein are differentially altered depending on the structural location of a mutation (Zimmermann et al., 2015), whether the mutation occurs at a domain interface, its intrinsic effect on thermodynamic stability of a domain (Auton et al., 2009), and/or its propensity for local disorder (Zimmermann et al., 2015). These intrinsic properties of the vWF triple A domain would therefore lead to different GroEL binding efficacies thus altering the urea denaturant pulse dependence of GroEL binding.

For V1369D, the disordered structure of the A1 domain (Tischer et al., 2014) is such that GroEL is able to bind to the vWF triple A domains even with low urea concentrations. By contrast, F1369I requires a much higher urea concentration to yield extensive GroEL binding. These observations imply that structural disorder induced in the A1 domain by these mutations result in altered quaternary A1-A2-A3 domain interactions that are differentially recognized by GroEL. The structural disruption of A1 by V1314D is so severe that GroEL readily recognizes exposed hydrophobic regions without urea denaturation. Conversely, F1369I, which also misfolds the A1 domain, may cause A1-A2-A3 domain to reorganize its quaternary structure forming unnatural domain interfaces which are stabilized against urea denaturation, thereby requiring higher urea to achieve similar levels of GroEL binding. This differential binding by GroEL depending on the mutation may be reduced by post-translational glycosylation, which normally decorates the vWF surface but are lacking when bacterially expressed.

The BLI denaturation pulse assay for the wild- and mutant-type proteins may have potential to be used as a rapid drug discovery platform. Performed with candidate small molecule stabilizers generated using *in silico* selection algorithms, this assay could be used to determine if the test compounds rectify the structural origins of misfolding. Any compound which returns proper folding to the mutant-type protein would return the mutant denaturation isotherm to match that for wild-type (Figure 4 arrows). Although these mutations presented herein are both in the A1 domain, a different stabilizing compound may be required to correct each specific mutation as the two mutants do not have the same effect on the denaturation isotherm and likely represent two different misfolding events which need to be stabilized.

The ability to generate kinetically controlled denaturation isotherms for entire sets of phenotypically distinct single site mutations may offer researchers a unique tool with the advantage of distinguishing properties of different mutant-types. A recent comparison of the wild type and mutant maltose binding protein constructs used in section Introduction also shows clearly different denaturation isotherms (Trecazzi and Fisher, 2018). For specific missense disease proteins, the observation of these kinetic differences in mutant stabilities indicate that one may have to design very specific correctors for specific mutations, strengthening the case for implementing personalized/precision medicine approaches to rectify both common and rare protein folding diseases.

## Transmission electron microscopy analysis of GroEL captured proteins

### Protein 3D reconstruction using cryoEM

Advances in obtaining atomic resolution structures using cryo-electron microscopy (cryoEM) still depend on sample integrity and purity (Earl et al., 2017). Before starting cryoEM imaging, it is imperative to assess sample integrity, which is usually accomplished by visualizing the sample using negative-stain EM. Optimal samples are those that are highly pure and homogenous with respect to conformation. Additionally, these negative-stained images can sometimes be used to generate preliminary 3D envelopes that can be useful to train automated particle picking programs. As with all negative-stain protein samples, there are caveats that must be considered, such as flattening and grid adherence effects, which in turn may lead to diminished conformational and orientation diversity. Despite these caveats, negative staining is still the much preferred first step in sample evaluation due to ease of preparation and relatively low cost. In solution, GroEL stabilizes aggregation prone proteins by temporarily removing misfolding species and preventing them from interacting with other proteins (Horwich et al., 2009; Tyagi et al., 2011). By pairing GroEL with the biosensors we can extend the use of this tool to study both the stability of target proteins and the location of the unfolding. The use of the chaperonin as both a capture-release and a capture scaffold platform that can facilitate visualization of the structures of aggregation-prone proteins using a very small quantity of sample will be discussed. The ability to immobilize and assemble complexes on the biosensor surface followed by release into microvolume drops permits determination of their structural integrity. Importantly, by demonstrating that it is possible to visualize proteins released from the GroEL biosensor surfaces, this capture-release technique enables one to potentially view conformations of aggregation prone proteins, a common problem that confronts many researchers who want to obtain credible 3D protein structures for their protein system of choice.

### GroEL stabilization of aggregation-prone proteins

#### GroEL-anthrax protective antigen stabilization

**Figure 5:**
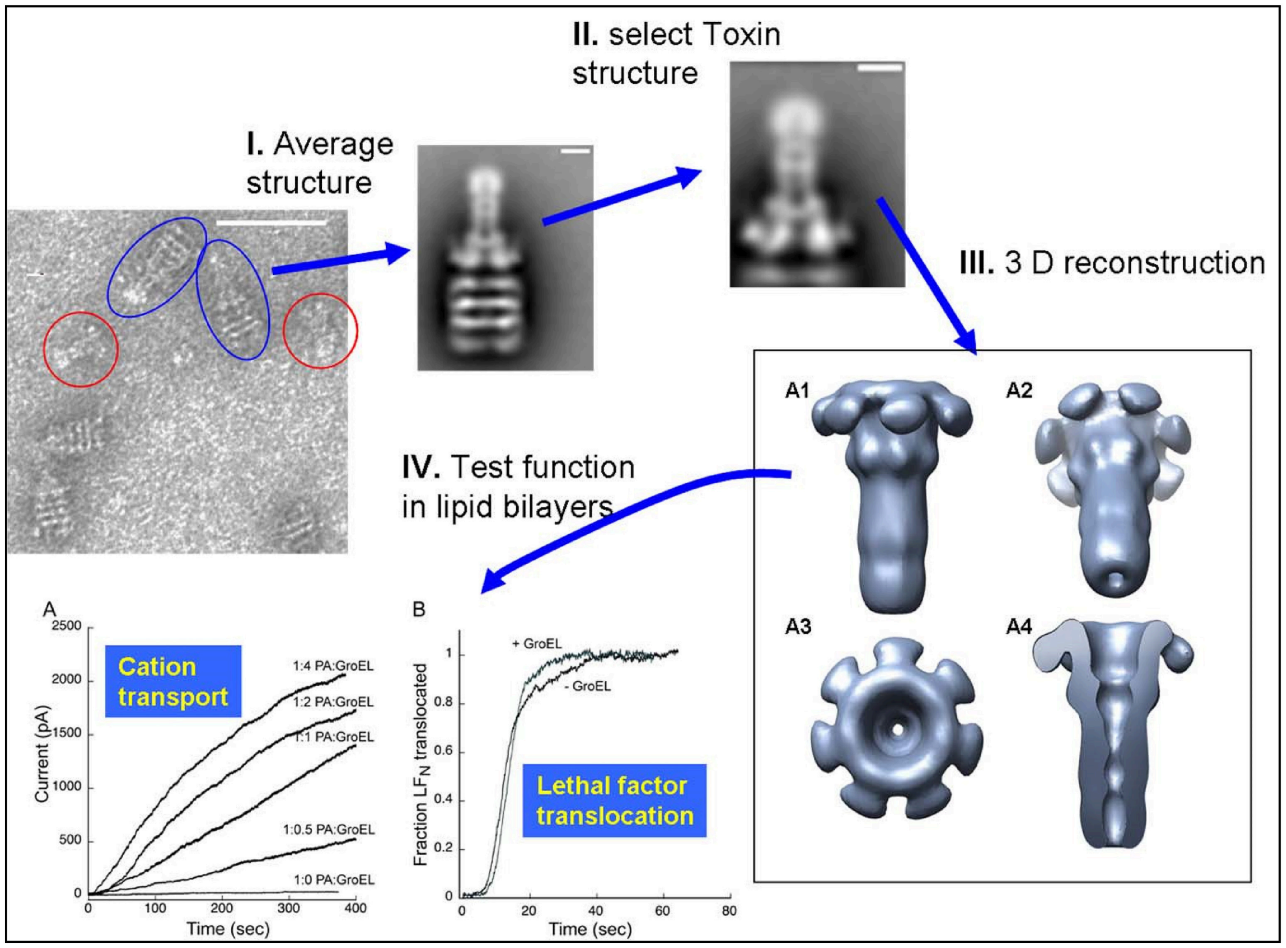
Schematic of the Protective Antigen Anthrax Pore Capture and Conversion. **(I)** 2D class computational averaging of GroEL-PA pore from the micrographs. **(II)** Masking of 2D class averages to reconstruct PA pore alone. **(III)** 3D envelope of isolated PA pore reconstructed using SPIDER, revealing the long β-barrel pore. **(IV)** PA pore functionality was as tested by both **(A)** cation transport and **(B)** lethal factor translocation assays. Micrographs obtained at UMKC. Adapted from Katayama et al. (2008).

The observation that the large 440 kDa protective antigen (PA) prepore structure can form stable complexes with the GroEL chaperonin in solution (see Figure 1 from Katayama et al., 2008) led to surmise GroEL-folded protein substrate complexes could also be visualized using EM. Thus, initial work from this laboratory confirmed that one can capture large folded protein complexes on the GroEL chaperonin and easily visualize these stable captured complexes using negative-stain EM. From these micrographs, low-resolution negative-stain reconstructions of the bound anthrax pore structure were obtained while simultaneously avoiding misfolding of this aggregation-prone toxin pore (see Figure 2 from Katayama et al., 2008). The compiled results from this analysis are summarized in Figure 5. These early results using GroEL as a molecular scaffold to capture the transitioned pore indicated that these low-resolution structures are quite similar in shape to the atomic resolution anthrax pore structures solved by Hong Zhou's group in 2015 (see Figure 1 from Jiang et al., 2015). In further agreement, domain 4 lacked electron density in both the atomic and the GroEL captured PA pore electron density maps, which is attributed to increased flexibility of this domain and not the limitation of the capture system. The formation of the complex between GroEL and the anthrax pore were driven primarily by electrostatic interactions and were easily reversed when ATP was added. In contrast to the physiological transition condition of low pH, the GroEL-PA prepore transition was accomplished by incubation in 1 M urea at 37°C. This condition is tolerated by both GroEL and GroEL-PA complex and is sufficient to permit the PA unfolding/refolding transition to occur while remaining bound to GroEL as a scaffold. This GroEL capture of the PA prepore allows successful transition from PA prepore to PA pore without allowing off-pathway aggregation, which predominates when PA transitions in solution. This is likely due to the fact that the GroEL-PA pore complex has a larger size (1.24 MDa) and, therefore, has a much slower diffusion rate as compared with the PA pore alone. Most importantly, this approach shows large macromolecular structures can be easily resolved while bound to GroEL. In the following sections, numerous examples are presented where this methodology is useful in aiding in the visualization of other folded proteins that are captured by the GroEL chaperonin.

#### Solution stabilization of tetanus neurotoxin

##### Tetanus neurotoxin

From the past 200 years of research into tetanus neurotoxin (TeNT), much about its functionality is known. However, the mechanistic details and the physical changes which accompany membrane insertion and protein transport across membranes under low pH conditions are unknown. To better understand this process, it is important to obtain the atomic structures of the neurotoxin upon neuron binding, membrane interface association, and membrane insertion via cryoEM. At the time that this manuscript was being assembled, a crystal structure and a cryoEM structure of tetanus neurotoxin was published (Masuyer et al., 2017). Even so, the negative-stain images of GroEL-TeNT and TeNT presented herein possess great similarity to the cryoEM TeNT structure with the 3D reconstructed electron density maps being in agreement. The following section highlights the ease by which this was accomplished and indicates that even a single resulting electron microscopy tilt series of the GroEL-TeNT complex exhibited discernable electron density surface topology that is highly similar to the cryoEM surface representation. In addition, it is demonstrated that a 3D reconstruction of TeNT can be generated using as few as 28 individual particles imaged as a tilt series.

##### GroEL prevents aggregation under low salt conditions

The previous three examples (MBP, IgG, & vWF) have demonstrated the use of GroEL in conjunction with BLI, but, as previously mentioned, GroEL can also be used in solution to prevent protein misfolding and aggregation. Once purified, TeNT is stored in a high salt solution (0.5 M NaCl) as it will aggregate under low salt conditions (0.05 M). However, aggregation of TeNT under low salt conditions can be prevented by addition of GroEL, presumably through the capture and release cycles of aggregation-prone, misfolded TeNT molecules. This permits visualization of TeNT using negative-stain EM, where the staining protocol becomes problematic due to high salinity.

##### Tilt series on the GroEL-stabilized TeNT

**Figure 6:**
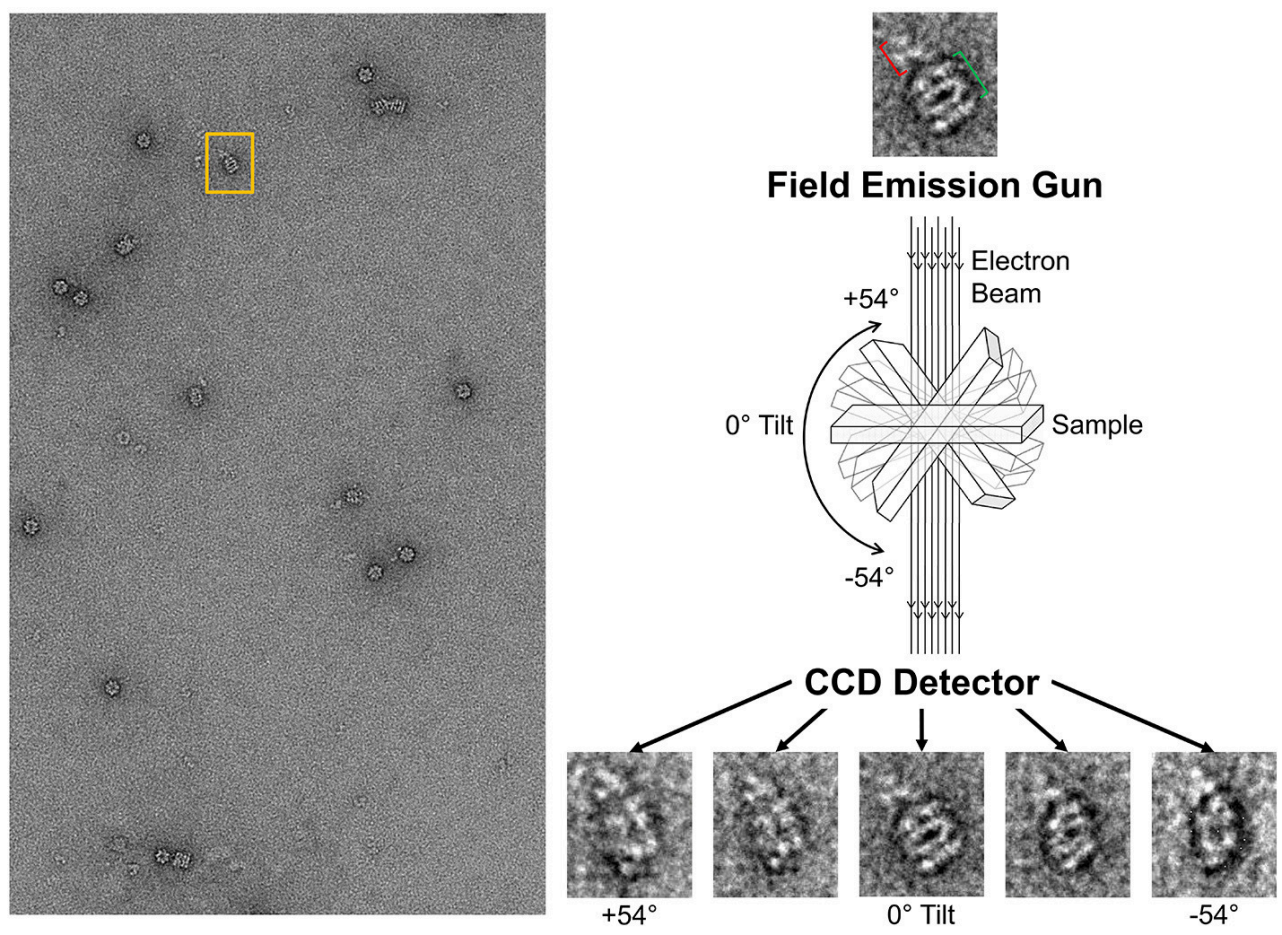
Schematic of Tilt Series Image Capture. **(Left)** Representative micrograph of negative-stained GroEL bound to TeNT. The grid is scanned for isolated complexes to avoid protein overlap at high tilt angles. An appropriate complex is boxed in orange. **(Right)** Enlargement of boxed complex for detail. GroEL can be seen as four parallel bands (green bracket). Captured TeNT (red bracket) can be seen as white density above GroEL. This complex is then repeatedly imaged at 2° increments. Representative tilted image captures are shown at the bottom. Micrographs obtained at MU EMC.

With the ability to visualize TeNT without aggregation, the gridded and stained TeNT can be imaged using a tilt enabled electron microscope and specimen holder. A tilt series was collected for this sample using a 200 keV field emission gun (Figure 6). A representative aligned tilt series is provided as a movie in Supplemental Video 1. One can clearly discern the cross like structure of the bound TeNT protein and the domain that interacts with GroEL appears to be the extended receptor binding domain. Comparison with the cryoEM single particle reconstruction envelope (EMD 3588) supports this conclusion. Supplied with only one single tilt series, 3D reconstruction of tetanus is not possible due to the limitation of the tilt angle preventing total conformation coverage. Additionally, as there is no preferred binding orientation between TeNT and GroEL, the pooling of images from multiple complexes does not result in a 3D envelope of the toxin likely due to differential GroEL capture of TeNT. This difficulty did not hamper the reconstruction of the GroEL-PA pore complex as the entire toxin was exclusively bound on top of the GroEL nanochamber as well as the complementary 7-fold symmetry match which aided to orient all PA pore in the same orientation relative to GroEL (Katayama et al., 2008). The reconstruction of the current complex is hindered by flattening effects due to dehydration and staining inherent in the negative-staining protocol. This flattening is evident by the GroEL appearing flat in the tilt series, lacking its characteristic barrel shape (Supplemental Video 1). This solution based reconstruction does not rule out the possibility of generating molecular structure for the GroEL-TeNT complex. Immobilization of TeNT in the same orientation may force GroEL binding at one site generating identical complexes. Orientation specific attachments have been engineered for TeNT previously (see discussion in Figure 10 in Lea et al., 2016).

##### TeNT reconstruction from negative-stain tilt series

**Figure 7:**
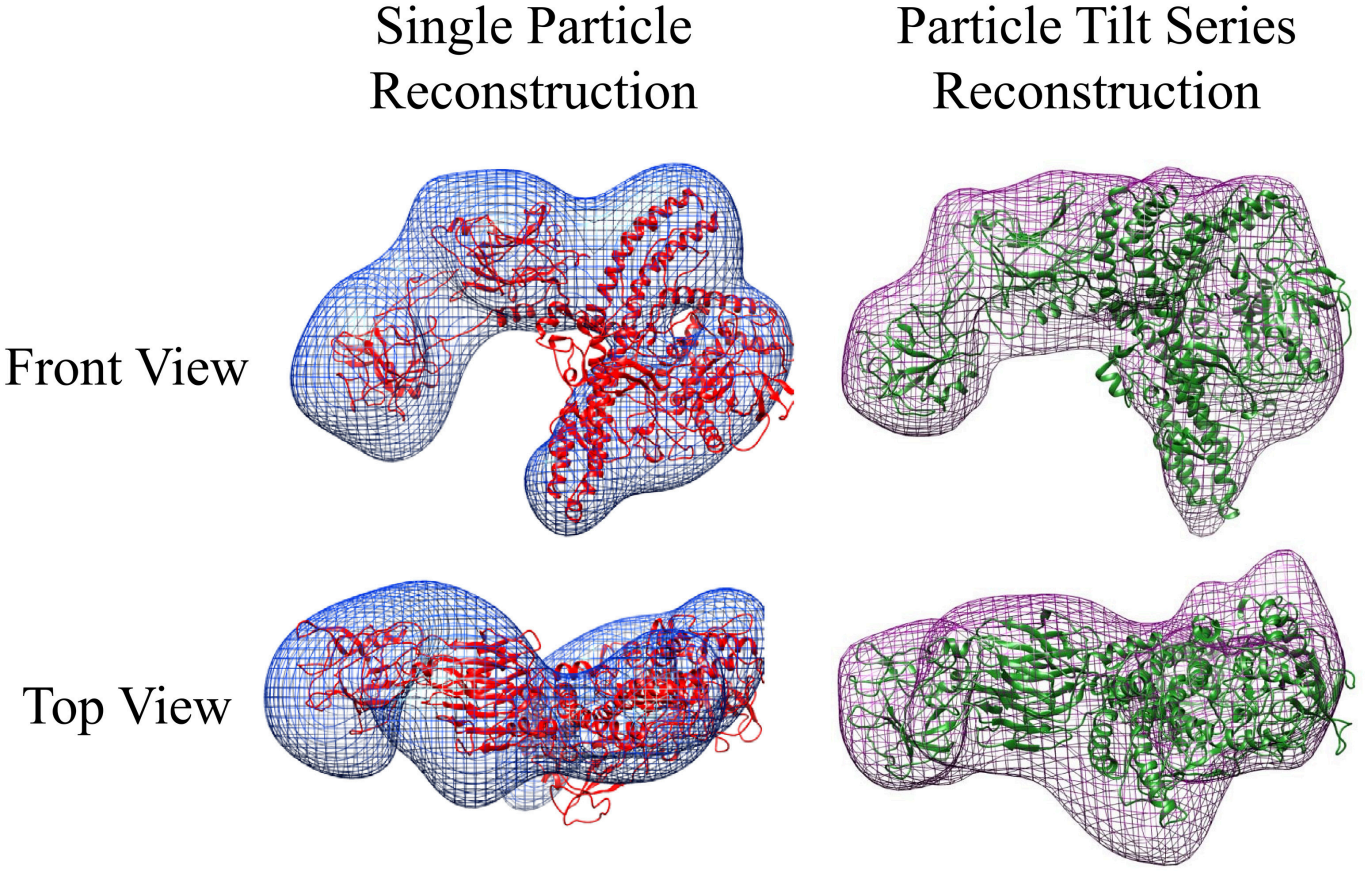
Negative-stain EM Comparative Reconstructions: Single Particle Reconstruction & Particle Tilt Series Reconstruction. **(Left)** Blue envelope of traditional single particle reconstruction using micrographs from Figure 6. Red ribbon structure of I-TASSER modeled protein fit using molecular dynamics into the envelope. **(Right)** Purple surface representation of reconstruction of 28 particles from tilt series acquisition. The green ribbon structure is the same I-TASSER fit into the map using MDFF. In this orientation, the receptor binding domain is placed on the left in each reconstruction.

Unlike the GroEL-TeNT complexes, the non-aggregated monomeric TeNT within the same field can be reconstructed using both single particle reconstruction and particle tilt series. Single particle reconstruction uses only the 0° tilts and hundreds of picked particles. In contrast, fewer particles can be used for reconstruction if the protein is repeatedly imaged while adjusting the tilt angle. The tilt angle is controlled by the acquisition software to ensure equal spacing of 2° between images. Using the cryoSPARC system, an electron density map can be constructed from only 28 unique particles (Figure 7; purple mesh, right). The movie for one aligned TeNT tilt series can be seen in Supplemental Video 2. This limited dataset reconstruction matches the traditional single particle reconstruction, which required >500 particles, (Figure 7; blue mesh, left) but did not require hundreds of particles to be picked. With further constraints on the input views as shown in Bartesaghi et al. (2012), higher resolution information could be obtained. The number of images generated from the tilt series of 28 particles yielded 1,500 views. Additionally, these two negative-stain reconstructions correlate, despite lower resolution, with the recently published work which solved the high resolution structure of TeNT by both cryoEM, created using 200,000 particles, and the SAXS ensemble (Masuyer et al., 2017). The resolution of these two negative-stain electron density maps can most certainly be improved by using higher tilt angles, increasing the number of particles picked, and ensuring the particles represent greater orientation coverage. However, the purpose of these initial reconstructions was not for high resolution but to demonstrate that low particle numbers can render reasonable low-resolution reconstructions using the cryoSPARC system. This approach may be very useful in situations where small amounts of purified complexes are assembled on BLI biosensors that are then released into microvolumes and visualized using negative-stain EM (see section Release and EM Analysis of Proteins Released From Biosensor Surfaces; Naik et al., 2013, 2014; Pace et al., 2018); thus, the reconstructions only achieved low-resolution. Although this molecular envelope is low-resolution (approximately 22 Å), the model can be used as a valid training dataset for automated neural network particle picking programs for both negative-stain EM and cryoEM micrographs.

### Release and EM analysis of proteins released from biosensor surfaces

As has been shown in sections Developing GroEL into a direct biosensor to detect partially folded protein populations within protein mixtures and Comparison of kinetically controlled denaturation isotherms of wild- and mutant-type von Willebrand Factor using GroEL-BLI denaturant pulse assay, the combination of BLI and GroEL is a powerful tool to assess protein stability. However, as BLI output is in nanometer shift in the interference spectrum and does not carry structural data, it requires the assumption that the expected protein complexes are being constructed on the biosensor surface. To validate complex assembly, the purported GroEL-protein complexes can be orthogonally confirmed using negative-stain EM. Using the proper conditions, the complexes formed in the previous sections can be released from the biosensors after complex assembly, and then gridded and stained with heavy metals (see section Materials and Methods for Experimental Details). Using this technique, the GroEL-protein complexes can be directly visualized. For other techniques that reveal high and low resolution election density envelops of protein complexes such as crystallography or small angle X-ray scattering respectively, it is ideal that the sample consists of homogeneous complexes. If the target protein is inherently varied with respect to conformational heterogeneity (multiple conformations), analyzing the structural outputs by these methods becomes problematic. With EM analysis, each complex can be examined individually and therefore the binding heterogeneity can potentially be revealed in each case, particularly if random tilt series methods are applied. While mass spectroscopy can also identify target protein composition upon ATP induced release from the GroEL chaperonin, the maintenance of solubility during EM analysis is critical. Including the natural anti-aggregation chaperonin protein allows one to obtain low resolution structures of both free and bound substrate protein chaperonin complexes. With GroEL-Protein substrate complexes, previous and new examples shown below indicate that direct EM visualization allows one to broadly identify which folded yet hydrophobic region(s) of the target protein interact with the GroEL chaperonin at its promiscuous protein substrate binding site.

#### Release of GroEL-vWF complexes from Ni-NTA biosensors

**Figure 8:**
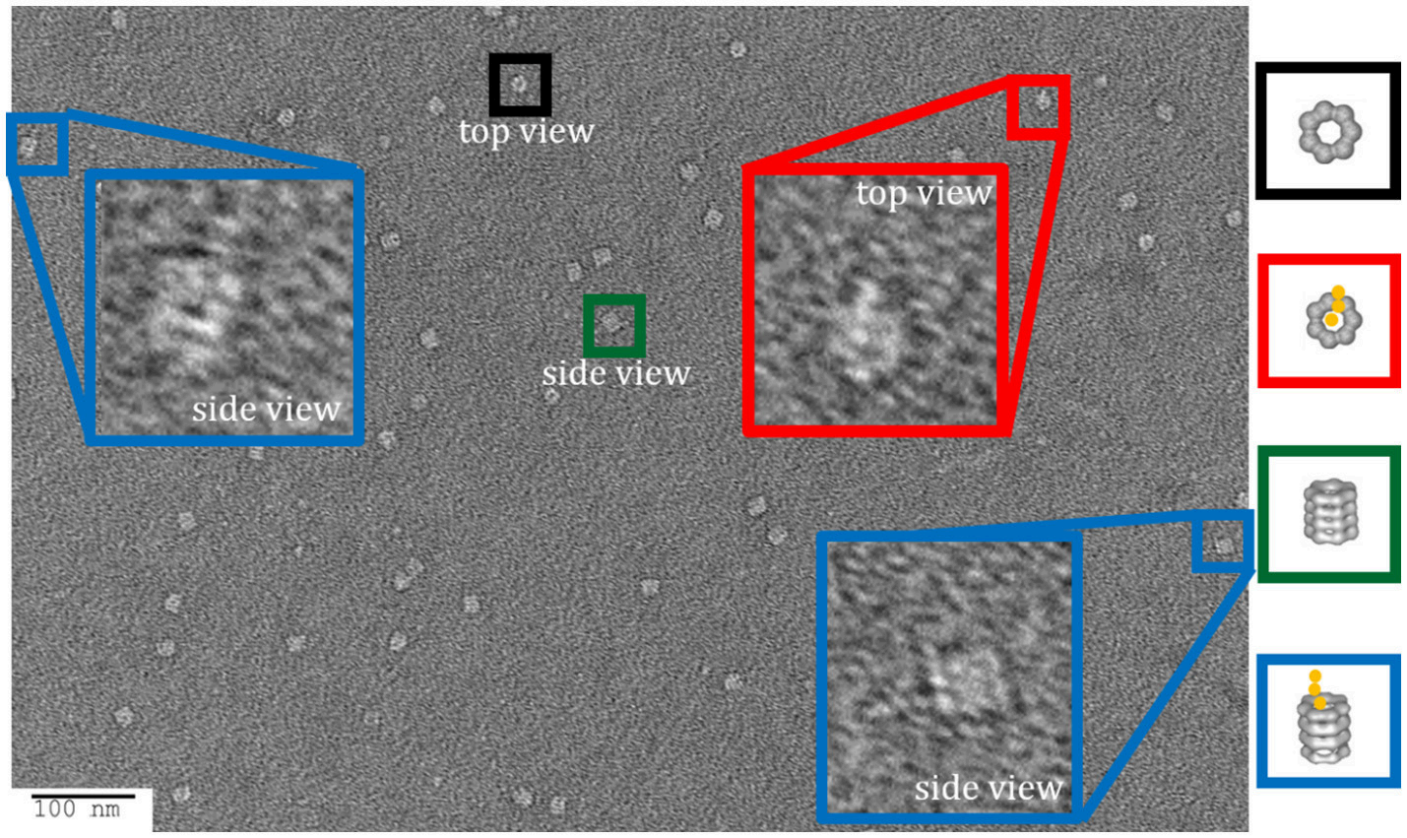
Imidazole Release of GroEL-vWF Complexes from Ni-NTA Biosensor. Representative field of gridded and stained V1314D vWF-GroEL complexes formed after a 2 M urea denaturant pulse. Multiple complexes can be seen as white on dark background. Top view of the complex is boxed in red, in which it is possible to observe the three domains of vWF. Side view of the complex is boxed in blue. Non-complexed GroEL top and side views are boxed in black and green, respectively. Micrographs obtained at KUMC.

For experiments where the GroEL-protein complexes were formed in the reverse orientation where the protein of interest is immobilized, like the vWF denaturation pulse assay presented section Comparison of kinetically controlled denaturation isotherms of wild- and mutant-type von Willebrand Factor using GroEL-BLI denaturant pulse assay of this paper, it is possible to visually confirm of the denaturant induced GroEL association. For experiments where the protein of interest has a His_6_ tag, the protein can be immobilized using Ni-NTA biosensors. The coordination between the His_6_ tag and the Ni^++^ ion can be gently reversed using either imidazole competition or EDTA chelation. It is imperative to optimize the eluent concentration and elution time using the BLI. By releasing captured proteins into a microvolume drop (3–4 μL), the concentration of any released protein will be relatively high and appropriate for EM. As an example of this release and visualization, the Ni-NTA tip used for the 2 M urea pulse on V1314D was released and stained for negative-stain EM. In this micrograph, it is possible to discern the distinct A1-A2-A3 domain extension, especially in the top view (Figure 8; red box). As the His_6_ tag is on the C-terminus of vWF, this orients the A3 domain closest to the biosensor surface and, therefore, sterically hindered against GroEL binding. Additionally, the previous solution equilibrium data indicates the A1 domain unfolds first (Auton et al., 2007a). With these two facts, it is most likely the A1 domain captured by GroEL. Although it is not possible to definitively identify the interacting domain with this sample, additional experiments could identify the interacting domains. For example, the addition of an anti-A1 antibody and its distinct density on the GroEL-vWF complex would help identify the GroEL interacting domain via negative-stain EM. In this particular field, the free GroEL observed is a consequence of not washing the biosensor before release. Extensive dissociation of the GroEL from vWF is not observed (see Figure 1 GroEL dissociation trace).

#### GroEL-IgG complexes released from GroEL biosensor surface

**Figure 9:**
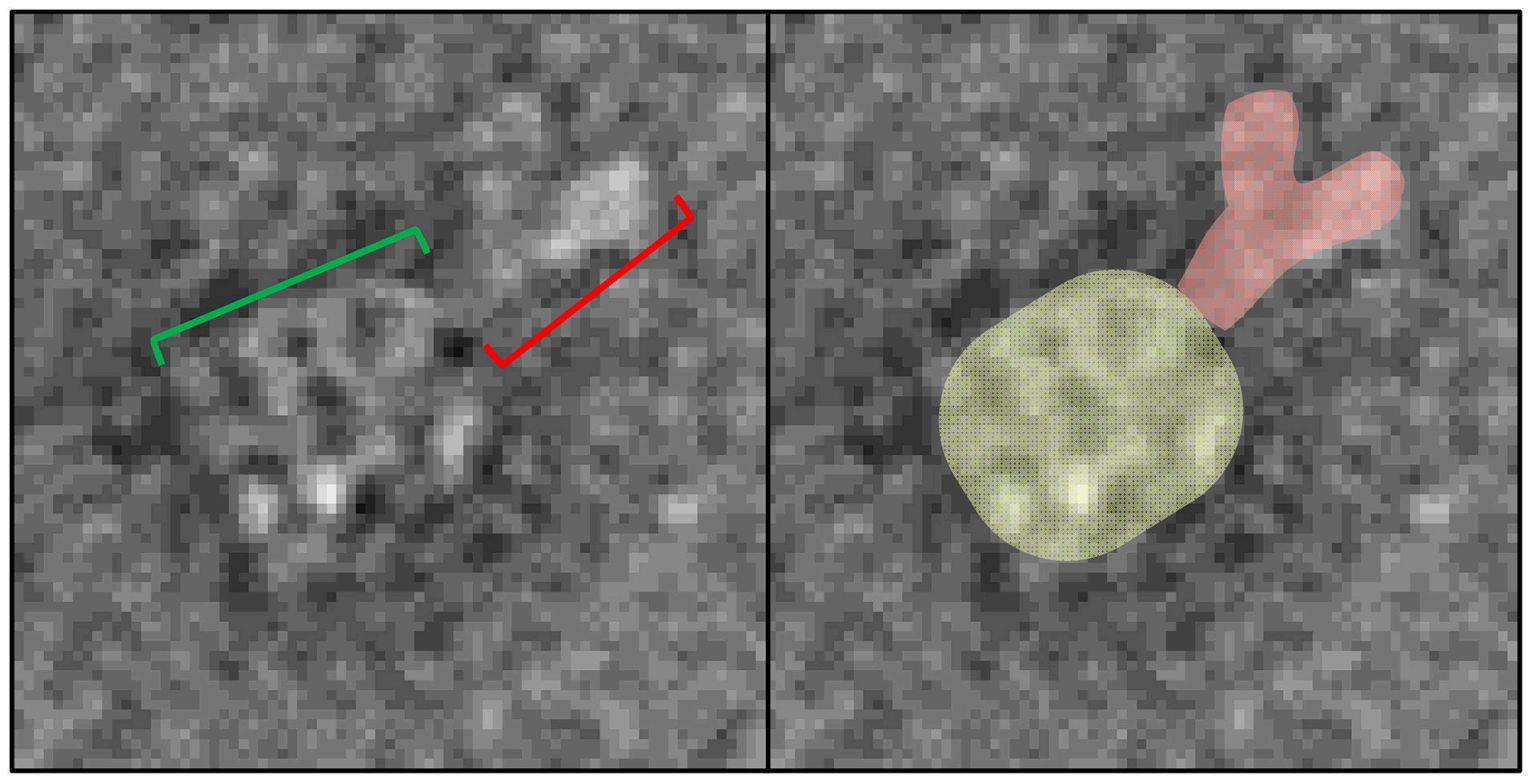
GroEL-IgG complex EM image. **(Left)** Representative particle of the DTT released complex from the modified bGroEL biosensor. The four parallel dark bars are GroEL (green bracket). The dark, extra density to the top right of GroEL is the captured IgG (red bracket). **(Right)** Same image where the proteins are false colored to aid in visualizing the protein boundaries. Images obtained at KUMC.

As shown in section Developing GroEL into a direct biosensor to detect partially folded protein populations within protein mixtures, GroEL biosensors are capable of detecting pre-aggregate transients that exist in solution before it is possible to observe larger scale aggregates using size exclusion chromatography or microflow imaging (Naik et al., 2014; Pace et al., 2018). In order to discern what region of IgG preferentially interact with GroEL, a cleavable biotinylation reagent needs to be employed so the GroEL-protein complex can be released. Replacing the standard biotinylation reagent with one containing a disulfide bond between the biotin moiety and the amine reactive group accomplishes this feat. Incubating the modified GroEL biosensor in 50 mM DTT will reduce the disulfide linkage to release GroEL-IgG complexes into solution. Similar to the imidazole release, optimization of the DTT incubation and release should be performed using BLI. Additionally, the same methodology for microvolume release and EM processing can be used as described above and previously (Naik et al., 2013, 2014; Pace et al., 2018). An example of this release is shown in Figure 9. The four, parallel dark bands are GroEL and the extra density to the top right of GroEL is the captured IgG (Naik et al., 2014). Although there was clear protein densities bound to GroEL in the 2014 Naik paper, upon further inspection of the micrographs there were individual EM images of the entire IgG molecule bound to GroEL. In views of the clearer, well-defined complexes, the IgG is bound to GroEL through its Fc portion of the antibody. This region is commonly found to be where aggregation prone antibodies display enhanced fluctuations (Pace et al., 2018).

Although the GroEL binding platform only indicates general regions where hydrophobic region exposure occurs on the Fc regions, the EM visualization of these GroEL-Ab complexes may also indicate aggregation prone regions (Pace et al., 2018). In some cases, these hydrophobic regions are pinpointed more precisely through the use of the more sensitive and elegant HDX assay. HDX examines which protein regions have changes in the dynamics and electrostatic interactions governing transient protein-protein interactions and finds them to be located within Fc regions (Majumdar et al., 2015; Arora et al., 2016, 2017). Of note, some these reversible interactions have been documented to be governed by electrostatic interactions. It is also possible that the ring of negative charge which surrounds the GroEL substrate binding site could also contribute to GroEL-Ab interactions. This notion, that some of these interactions may be electrostatic in nature, has not been specifically tested. This could be easily determined by adjusting the ionic strength of the solution or by adding in positively charged species such as arginine.

#### Release of GroEL captured aggregation prone proteins from biosensor surfaces

**Figure 10:**
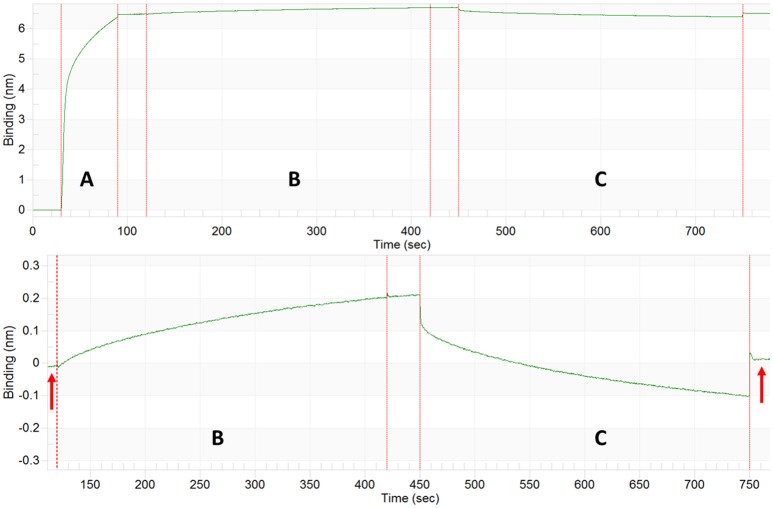
Tetanus Neurotoxin Capture and ATP Release by GroEL Biosensor. Top: BLI sensogram indicating biotinylated GroEL association **(A)**, TeNT capture by the GroEL biosensor **(B)**, and ATP release of TeNT from GroEL biosensor **(C)**. Bottom: Enlargement of TeNT capture and release steps to highlight amplitude change for capture and release. The baselines before association and after ATP dissociation match indicating that the signal increase and decrease are solely from GroEL capturing and releasing TeNT (red arrows). The large initial shifts in the trace for the ATP phase **(C)** are due to a refractive index changes and not a change in protein binding.

**Figure 11:**
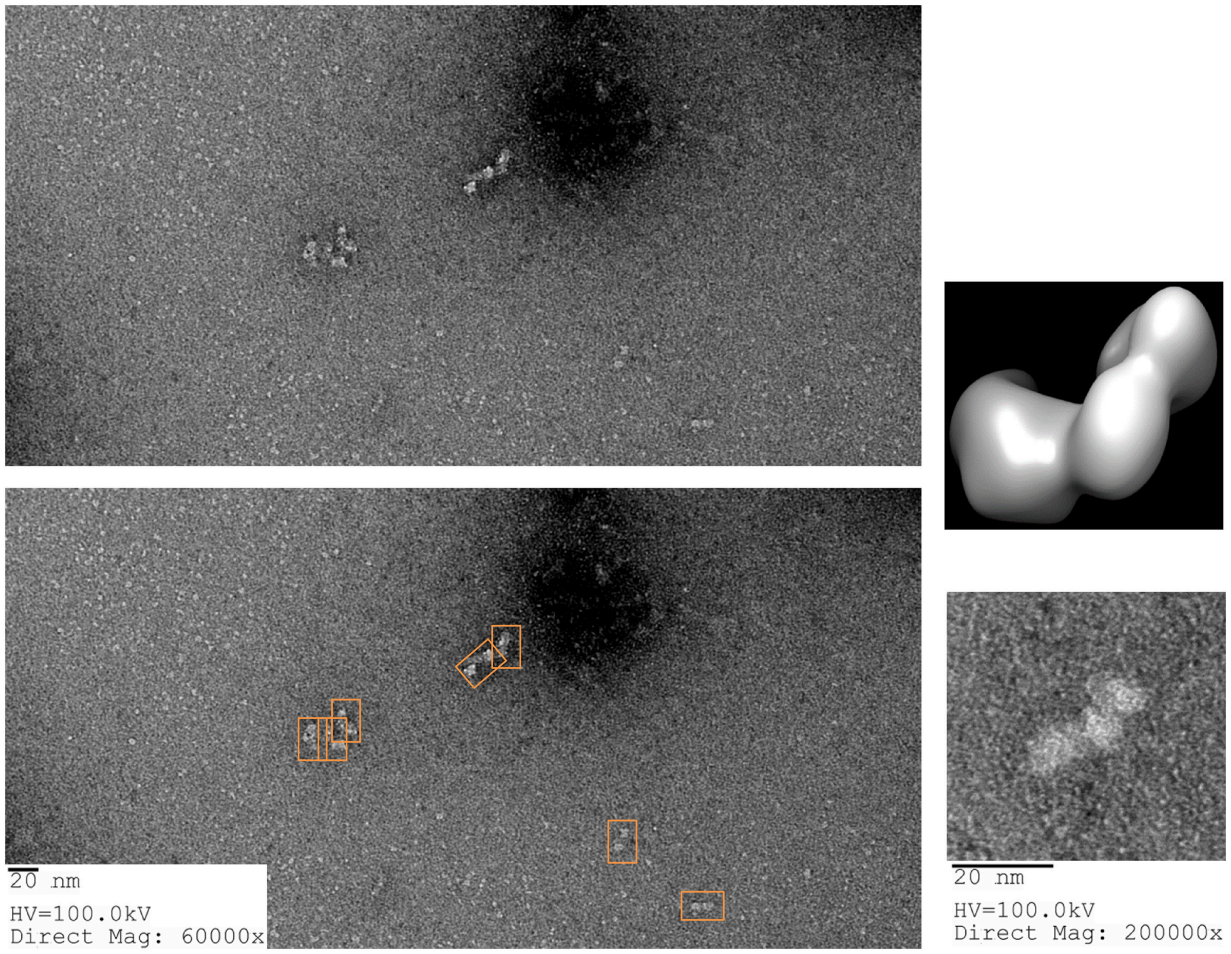
ATP Release of Tetanus Neurotoxin from GroEL Biosensor. **(Left)** Representative field of gridded and stained TeNT after release from GroEL biosensor using ATP. Multiple copies of the protein can be seen as white on dark background (orange boxes on the duplicate image below). **(Right)** Higher magnification of isolated TeNT molecules. In the magnified field, the three domains of tetanus can be observed. For comparison, the envelope and ribbon structure from Figure 7 have been oriented in the same view as the particle and recolored to match the white on black of the EM image. Images obtained at KUMC.

For the GroEL biosensors, one can exploit the native functions of the chaperonin to release the captured proteins for visualization. As previously stated, GroEL can hydrolyze ATP to initiate folding and subsequent release of substrate proteins from GroEL. If the GroEL biosensor is introduced to an ATP solution, the captured proteins will be released into solution. As previously, the microvolume method and optimization by BLI should be utilized. An example using TeNT as the protein substrate is given in Figure 10. As can be seen in the sensogram, 10 mM ATP achieves nearly full release within 5 min. The experiment can be repeated in the microvolume drop and prepared for EM imaging (Figure 11). The enlarged fields show the distinct three domain structure of TeNT.

## Conclusion

The GroE chaperonin system has a distinct advantage that the nucleotide-free state of the large chaperonin oligomer maintains a constant hydrophobic surface that can capture transient or existing solvent exposed hydrophobic patches. This fact offers a superior level of detection capabilities for partially folded states that are unique to this particular chaperone. While other protein chaperone classes such as the Hsp90, 70 and small Hsp proteins exhibit similar global recognition properties of hydrophobicity, the GroEL chaperonin class (Hsp60) has a high affinity substrate capture state that is more promiscuous than other systems. Most importantly, specific binding is easily reversed. Although there are many reviews indicating the absolute requirement of the Hsp10 co-chaperonin class for release of more stringent substrates, our laboratory has found that this requirement is not necessary if folding osmolytes or osmolyte mixtures are included along with ATP to reverse binding to the chaperonin (Voziyan et al., 2000; Voziyan and Fisher, 2002; Fisher and Katayama, 2015). With regard to the other chaperone classes, the Hsp90 and Hsp70 proteins exhibit various binding states for hydrophobic proteins, but their nucleotide-free forms are often not the highest affinity capture state. In the Hsp90 class, there are a number of co-chaperone accessory proteins that are required for substrate specificity and binding. The small heat shock protein class certainly binds to hydrophobic regions to sequester aggregation-prone proteins, and so they have been termed holdases. However, these small heat shock proteins require other chaperone protein systems such as the Hsp70 system to facilitate release and folding (Zwirowski et al., 2017). For other Hsp families, such as the Hsp100 class, the oligomeric forms, which bind to protein aggregates, are not stable as these chaperone proteins assemble and disassemble in a nucleotide dependent manner (Zolkiewski et al., 1999). From the standpoint of constructing immobilized chaperone biosensor platforms, the above-mentioned chaperone classes for the most part have to be specifically oriented on the biosensor surface to properly position the one prominent substrate binding site on these Hsp classes. In contrast, the GroEL chaperonin system is much easier to immobilize as it possesses two opposing binding sites. Even random immobilization schemes always result in the exposure of at least one binding site.

Based on the properties of the chaperonin, there are a few limitations that may impair the utility of the GroEL based detection platforms. The simplest limitation for chaperonin based detection of partially folded substrates depends on the surface electrostatic field that surrounds the chaperonin binding site (Coyle et al., 1997). For example although the chaperonin readily binds to neural folding disease proteins such as Aβ amyloid and α synuclein (Ojha et al., 2016; O'Neil et al., 2018) the chaperonin was unable to bind to aggregation prone tau perhaps due to electrostatic repulsion effects (preliminary data). Indeed, electrostatic effects on binding were also demonstrated with the anthrax toxin (discussed above in section GroEL-anthrax protective antigen stabilization) In this work, we present the use of GroEL and biosensors together to distinguish differential kinetic binding and stability differences that can be observed with between wild type and mutant variants of two proteins, MBP and vWF. Although we have previously demonstrated that the chaperonin capture platform can distinguish between native and mutant forms of various other mutant proteins (Naik et al., 2010 – wild type and mutant transthyretin mutants; Correia et al., 2014 – wild type and mutants of frataxin), the previous chaperonin bead-based methods were tedious and cumbersome. The biosensor denaturant pulse approach applied with the vWF variants shows that this type of comparative analysis easily highlight kinetic stabilities differences between wild type and various mutant forms and most importantly, dramatically accelerates mutant comparison analysis. The GroEL biosensor is based upon the GroEL chaperonin and its properties, namely its promiscuous nature to bind and capture any exposed hydrophobic patches. Therefore, the GroEL biosensor should bind to any target protein with exposed regions. Since mutant hydrophobic surfaces can be variable (e.g., different m values from chemical denaturation profiles), the kinetic partitioning binding reactions of the chaperonin onto the target protein biosensor results in distinct kinetic profiles. We can certainly envision instances where mutant vs. wild type comparisons may fail, seen previous for protein fragment comparisons (e.g., CFTR NDB1 in Lea et al., 2016). For detecting mutant populations in solution using the chaperonin biosensor approach, rapid aggregation of mutants will also interfere with one's ability to clearly differentiate between mutant classes. In addition, there could also be instances where mutant and wild type proteins may naturally exist in kinetically destabilized states (i.e., intrinsically disordered regions). In this latter case, the chaperonin biosensor systems may not be able to distinguish between these two dynamic states.

In the first set of experiments, the prospect of analyzing protein mixtures with the chaperonin system was explored. This approach is particularly intriguing given the wide range of alterations in protein integrity that can lead to enhanced downstream aggregation. The GroEL biosensor systems are useful in discriminating between stable and dynamically unstable species that can exist within one protein solution. To expand on this approach, it may be useful to assess the degree of possible chemical modification of protein populations that result in destabilization, resulting in more aggregation prone species. These altered protein populations can then be released from the GroEL biosensor and analyzed by highly sensitive mass spectroscopy methods. To further enhance the chaperonin detection methodologies, it will be useful to develop bulk approaches where larger concentrations of chaperonins are used to interact with the bulk system rather than a smaller biosensor. Indeed, bulk type experiments have been used to capture entire dynamic transient protein systems that rely on insuring that the chaperonin protein is present in excess of the target protein (Smith et al., 1998; Correia et al., 2014).

However, it is important to reemphasize how the chaperonin biosensor approaches can dramatically accelerate the detection of these transient dynamic states that exist in solution over the above mentioned bulk methods. This development is particularly relevant in that it provides rapid solution-based kinetic analysis that can even complement other more structurally intensive evaluations of protein dynamics such as hydrogen/deuterium exchange mass spectroscopy methods. For example, our previous evaluations of examining the protein stability of particular IgG molecules (Pace et al., 2018) both with biosensor partitioning kinetics and examining GroEL-IgG complexes by electron microscopy are orthogonally supported by comprehensive HD exchange mapping of dynamic regions within same antibody samples (Toth et al., 2018). It will be particularly interesting to compare the association kinetics of protein solutions that contain dynamically fluctuating populations of native and partially unfolded populations with global HD exchange kinetic profiles. For example, the association kinetics of proteins partitioning onto chaperonin biosensors contain multiple kinetic phases where in some instances, a burst in association kinetics is followed by a slower steady rise in signal (Figure 3). This burst kinetics profile followed by a slow steady rise in signal is also observed in global HD exchange kinetic outputs (Yan and Maier, 2009).

While the mesophilic GroEL chaperonin system isolated from *Escherichia coli* is broadly applicable in monitoring the stability of target proteins using the denaturant pulse experimental platform used in the second experimental section, it may be very interesting to expand the denaturation conditions to include those that are sometimes encountered in cellular environments. For example, the *E. coli* chaperonin exhibits a broad stability range across temperature and pH as assessed by various structural monitors (Naik et al., 2014). It will be intriguing to determine if this stability range can be expanded or dramatically shifted by using chaperonin systems isolated from extremophiles. It may be possible to assess the cold stability of a target protein using a chaperonin isolated from psychrophilic organisms in a similar denaturation platform. Psychrophilic chaperonins are often used in bacterial expression system to help fold proteins that have a tendency to aggregate at higher temperatures. Likewise, chaperonins from thermophilic, acidophilic, barophilic, and halophilic organisms may be useful to expand the denaturation conditions of target protein to include higher temperatures, acidic conditions, high hydrostatic pressures, and high ionic strength solutions. Similar to the *E. coli* chaperonin, these extremophile systems will likely be able to detect the pre-aggregate states of target proteins. The detection of pre-aggregate states are much more rapid and most importantly, detection occurs before large scale aggregation takes places. Popular evaluation aggregation propensity depends on techniques such as microflow imaging and size exclusion chromatography where these methods rely on separation or direct visualization of time dependent increases in molecular weight (Pace et al., 2018). GroEL biosensors, on the other hand, appear to detect the formation of transient or long-lived hydrophobic patches on monomeric states.

Finally, others have begun to use chaperonins as both capture platforms and enhanced structural probes for electron microscopy to probe the nature of protein aggregation reactions. Recently, G. M. Clore's group used the *E. coli* chaperonin protein to decorate prion polymers to enhance visualization of hydrophobic patches using EM analysis. What was particular striking from the image analysis in this work was the observation that GroEL bound to the extended fibular arrays in a surprisingly discernable repeating pattern. Given that the chaperonin is primarily binding hydrophobic patches, it is logical to conclude that regular hydrophobic patterns exist within these aggregated arrays (Wälti et al., 2017). In a related approach, the demonstration that large proteins can bind to the chaperonin binding site may be useful in pinpointing specific aggregation prone regions if enough complexes can be formed for reconstruction analysis, particularly if one simply focuses on the interaction regions exclusively without including more flexible regions (Naik et al., 2014; Pace et al., 2018; Figure 9 this work).

The data presented herein illustrates the broad utility of using the promiscuous chaperonin to (1) capture kinetic transients, (2) distinguish various mutant-type folds, and (3) enhance structure assessment of large proteins using electron microscopy. All of these applications arose from the simple observation that the binding affinity of some folding proteins leads to folding arrest and long-term sequestration of protein substrates. As noted above, it will be interesting to expand the role of chaperonin capture and release strategies to examine initial structural stages of protein aggregation, a truly elusive reaction time regime that may provide enormous benefits in understanding the molecular basis of some human protein folding diseases.

## Author contributions

MF, PO, and AM conceived and designed the experiments. PO, AM, BD, and TW performed the experiments. PO, AM, BD, and CT analyzed the data. PO and CT performed computation. CT acquired and installed computational tools and necessary libraries. MF, PO, AM, and BD wrote the manuscript. PO, MF, AM, CT, AT, MA, VM, MB, and TW edited the manuscript. PO, BD, CT, AT, VM, MA, and MB, purified protein.

### Conflict of interest statement

The authors declare that the research was conducted in the absence of any commercial or financial relationships that could be construed as a potential conflict of interest.

## References

[B1] Akkaladevi N. Mukherjee S. Katayama H. Janowiak B. Patel D. Gogol E. P.. (2015). Following natures lead: on the construction of membrane-inserted toxins in lipid bilayer nanodiscs. J. Membr. Biol. 248, 595–607. 10.1007/s00232-014-9768-3 25578459 PMC4580227

[B2] Arora J. Hu Y. Esfandiary R. Sathish H. A. Bishop S. M. Joshi S. B.. (2016). Charge-mediated Fab-Fc interactions in an IgG1 antibody induce reversible self-association, cluster formation, and elevated viscosity. MAbs 8, 1561–1574. 10.1080/19420862.2016.1222342 27560842 PMC5098451

[B3] Arora J. Joshi S. B. Middaugh C. R. Weis D. D. Volkin D. B. (2017). Correlating the effects of antimicrobial preservatives on conformational stability, aggregation propensity, and backbone flexibility of an IgG1 mAb. J. Pharm. Sci. 106, 1508–1518. 10.1016/j.xphs.2017.02.007 28212986

[B4] Auton M. Cruz M. A. Moake J. (2007a). Conformational stability and domain unfolding of the von willebrand factor a domains. J. Mol. Biol. 366, 986–1000. 10.1016/j.jmb.2006.10.067 17187823

[B5] Auton M. Holthauzen L. M. Bolen D. W. (2007b). Anatomy of energetic changes accompanying urea-induced protein denaturation. Proc. Natl. Acad. Sci. U.S.A. 104, 15317–15322. 10.1073/pnas.0706251104 17878304 PMC2000523

[B6] Auton M. Sedlák E. Marek J. Wu T. Zhu C. Cruz M. A. (2009). Changes in thermodynamic stability of von Willebrand factor differentially affect the force-dependent binding to platelet GPIbalpha. Biophys. J. 97, 618–627. 10.1016/j.bpj.2009.05.009 19619477 PMC2711320

[B7] Auton M. Sowa K. E. Smith S. M. Sedlák E. Vijayan K. V. Cruz M. A. (2010). Destabilization of the A1 domain in von Willebrand factor dissociates the A1A2A3 tri-domain and provokes spontaneous binding to glycoprotein Ibalpha and platelet activation under shear stress. J. Biol. Chem. 285, 22831–22839. 10.1074/jbc.M110.103358 20498367 PMC2906274

[B8] Bartesaghi A. Lecumberry F. Sapiro G. Subramaniam S. (2012). Protein secondary structure determination by constrained single-particle cryo-electron tomography. Structure 20, 2003–2013. 10.1016/j.str.2012.10.016 23217682 PMC3600145

[B9] Burns J. R. Baldwin M. R. (2014). Tetanus neurotoxin utilizes two sequential membrane interactions for channel formation. J. Biol. Chem. 289, 22450–22458. 10.1074/jbc.M114.559302 24973217 PMC4139251

[B10] Chapman E. Farr G. W. Usaite R. Furtak K. Fenton W. A. Chaudhuri T. K.. (2006). Global aggregation of newly translated proteins in an *Escherichia coli* strain deficient of the chaperonin GroEL. Proc. Natl. Acad. Sci. U.S.A. 103, 15800–15805. 10.1073/pnas.0607534103 17043235 PMC1613232

[B11] Clark A. C. Ramanathan R. Frieden C. (1998). Purification of GroEL with low fluorescence background. Meth. Enzymol. 290, 100–118. 10.1016/S0076-6879(98)90010-6 9534154

[B12] Correia A. R. Naik S. Fisher M. T. Gomes C. M. (2014). Probing the kinetic stabilities of Friedreich's ataxia clinical variants using a solid phase GroEL chaperonin capture platform. Biomolecules 4, 956–979. 10.3390/biom4040956 25333765 PMC4279165

[B13] Coyle J. E. Jaeger J. Gross M. Robinson C. V. Radford S. E. (1997). Structural and mechanistic consequences of polypeptide binding by GroEL. Fold. Des. 2, R93–R104. 10.1016/S1359-0278(97)00046-1 9427006

[B14] Earl L. A. Falconieri V. Milne J. L. Subramaniam S. (2017). Cryo-EM: beyond the microscope. Curr. Opin. Struct. Biol. 46, 71–78. 10.1016/j.sbi.2017.06.002 28646653 PMC5683925

[B15] Fisher M. T. (1993). On the assembly of dodecameric glutamine synthetase from stable chaperonin complexes. J. Biol. Chem. 268, 13777–13779. 8100224

[B16] Fisher M. T. (1998). GroE chaperonin-assisted folding and assembly of dodecameric glutamine synthetase. Biochemistry Mosc. 63, 382–398. 9556521

[B17] Fisher M. T. Katayama H. (2015). Osmolyte Mixture for Protein Stabilization. Patent # 61/237,451 United States Patent and Trademark Office.

[B18] Fisher M. T. Yuan X. (1994). The rates of commitment to renaturation of rhodanese and glutamine synthetase in the presence of the groE chaperonins. J. Biol. Chem. 269, 29598–29601. 7961947

[B19] Gruber R. Horovitz A. (2016). Allosteric mechanisms in chaperonin machines. Chem. Rev. 116, 6588–6606. 10.1021/acs.chemrev.5b00556 26726755

[B20] Horwich A. L. Apetri A. C. Fenton W. A. (2009). The GroEL/GroES cis cavity as a passive anti-aggregation device. FEBS Lett. 583, 2654–2662. 10.1016/j.febslet.2009.06.049 19577567 PMC2759771

[B21] Jiang J. Pentelute B. L. Collier R. J. Zhou Z. H. (2015). Atomic structure of anthrax protective antigen pore elucidates toxin translocation. Nature 521, 545–549. 10.1038/nature14247 25778700 PMC4519040

[B22] Katayama H. Janowiak B. E. Brzozowski M. Juryck J. Falke S. Gogol E. P.. (2008). GroEL as a molecular scaffold for structural analysis of the anthrax toxin pore. Nat. Struct. Mol. Biol. 15, 754–760. 10.1038/nsmb.1442 18568038 PMC2504863

[B23] Keeney S. Cumming A. M. (2001). The molecular biology of von Willebrand disease. Clin. Lab. Haematol. 23, 209–230. 10.1046/j.1365-2257.2001.00400.x 11683782

[B24] Kremer J. R. Mastronarde D. N. McIntosh J. R. (1996). Computer visualization of three-dimensional image data using IMOD. J. Struct. Biol. 116, 71–76. 10.1006/jsbi.1996.0013 8742726

[B25] Lea W. A. O'Neil P. T. Machen A. J. Naik S. Chaudhri T. McGinn-Straub W.. (2016). Chaperonin-based biolayer interferometry to assess the kinetic stability of metastable, aggregation-prone proteins. Biochemistry 55, 4885–4908. 10.1021/acs.biochem.6b00293 27505032 PMC5524994

[B26] Llorca O. Galán A. Carrascosa J. L. Muga A. Valpuesta J. M. (1998). GroEL under heat-shock. Switching from a folding to a storing function. J. Biol. Chem. 273, 32587–32594. 10.1074/jbc.273.49.32587 9829996

[B27] Machha V. R. Tischer A. Moon-Tasson L. Auton M. (2017). The von willebrand factor A1-collagen III interaction is independent of conformation and type 2 von willebrand disease phenotype. J. Mol. Biol. 429, 32–47. 10.1016/j.jmb.2016.11.014 27889474 PMC5186406

[B28] Majumdar R. Middaugh C. R. Weis D. D. Volkin D. B. (2015). Hydrogen-deuterium exchange mass spectrometry as an emerging analytical tool for stabilization and formulation development of therapeutic monoclonal antibodies. J. Pharm. Sci. 104, 327–345. 10.1002/jps.24224 25354868

[B29] Martin J. Horwich A. L. Hartl F. U. (1992). Prevention of protein denaturation under heat stress by the chaperonin Hsp60. Science 258, 995–998. 1359644 10.1126/science.1359644

[B30] Masuyer G. Conrad J. Stenmark P. (2017). The structure of the tetanus toxin reveals pH-mediated domain dynamics. EMBO Rep. 18, 1306–1317. 10.15252/embr.201744198 28645943 PMC5538627

[B31] Naik S. Brock S. Akkaladevi N. Tally J. McGinn-Straub W. Zhang N.. (2013). Monitoring the kinetics of the pH-driven transition of the anthrax toxin prepore to the pore by biolayer interferometry and surface plasmon resonance. Biochemistry 52, 6335–6347. 10.1021/bi400705n 23964683 PMC3790466

[B32] Naik S. Haque I. Degner N. Kornilayev B. Bomhoff G. Hodges J.. (2010). Identifying protein stabilizing ligands using GroEL. Biopolymers 93, 237–251. 10.1002/bip.21319 19802819 PMC2805906

[B33] Naik S. Kumru O. S. Cullom M. Telikepalli S. N. Lindboe E. Roop T. L.. (2014). Probing structurally altered and aggregated states of therapeutically relevant proteins using GroEL coupled to bio-layer interferometry. Protein Sci. 23, 1461–1478. 10.1002/pro.2515 25043635 PMC4287005

[B34] Ojha B. Fukui N. Hongo K. Mizobata T. Kawata Y. (2016). Suppression of amyloid fibrils using the GroEL apical domain. Sci. Rep. 6:31041. 10.1038/srep31041 27488469 PMC4973282

[B35] O'Neil P. T. Machen A. J. Thompson J. A. Wang W. Hoang Q. Q. Baldwin M. R. (2018). Constructing kinetically controlled denaturation isotherms of folded proteins using denaturant-pulse chaperonin binding. Methods Mol. Biol. 55, 4885–4908. 10.1007/978-1-4939-8820-4_19 PMC6196737 30341618

[B36] Pace S. E. Joshi S. B. Esfandiary R. Stadelman R. Bishop S. M. Middaugh C. R.. (2018). The use of a GroEL-BLI biosensor to rapidly assess preaggregate populations for antibody solutions exhibiting different stability profiles. J. Pharm. Sci. 107, 559–570. 10.1016/j.xphs.2017.10.010 29037466

[B37] Punjani A. Rubinstein J. L. Fleet D. J. Brubaker M. A. (2017). cryoSPARC: algorithms for rapid unsupervised cryo-EM structure determination. Nat. Methods 14, 290–296. 10.1038/nmeth.4169 28165473

[B38] Roberts C. J. (2014). Protein aggregation and its impact on product quality. Curr. Opin. Biotechnol. 30, 211–217. 10.1016/j.copbio.2014.08.001 25173826 PMC4266928

[B39] Saibil H. R. Fenton W. A. Clare D. K. Horwich A. L. (2013). Structure and allostery of the chaperonin GroEL. J. Mol. Biol. 425, 1476–1487. 10.1016/j.jmb.2012.11.028 23183375

[B40] Sewell B. T. Best R. B. Chen S. Roseman A. M. Farr G. W. Horwich A. L.. (2004). A mutant chaperonin with rearranged inter-ring electrostatic contacts and temperature-sensitive dissociation. Nat. Struct. Mol. Biol. 11, 1128–1133. 10.1038/nsmb844 15475965

[B41] Smith K. E. Fisher M. T. (1995). Interactions between the GroE chaperonins and rhodanese. Multiple intermediates and release and rebinding. J. Biol. Chem. 270, 21517–21523. 10.1074/jbc.270.37.21517 7665563

[B42] Smith K. E. Voziyan P. A. Fisher M. T. (1998). Partitioning of rhodanese onto GroEL. Chaperonin binds a reversibly oxidized form derived from the native protein. J. Biol. Chem. 273, 28677–28681. 10.1074/jbc.273.44.28677 9786862

[B43] Tang G. Peng L. Baldwin P. R. Mann D. S. Jiang W. Rees I.. (2007). EMAN2: an extensible image processing suite for electron microscopy. J. Struct. Biol. 157, 38–46. 10.1016/j.jsb.2006.05.009 16859925

[B44] Tischer A. Machha V. R. Frontroth J. P. Brehm M. A. Obser T. Schneppenheim R.. (2017). Enhanced local disorder in a clinically elusive von willebrand factor provokes high-affinity platelet clumping. J. Mol. Biol. 429, 2161–2177. 10.1016/j.jmb.2017.05.013 28533135 PMC5839150

[B45] Tischer A. Madde P. Moon-Tasson L. Auton M. (2014). Misfolding of vWF to pathologically disordered conformations impacts the severity of von Willebrand disease. Biophys. J. 107, 1185–1195. 10.1016/j.bpj.2014.07.026 25185554 PMC4156683

[B46] Toth R. T. IV. Pace S. E. Mills B. J. Joshi S. B. Esfandiary R. Middaugh C. R.. (2018). Evaluation of hydrogen exchange mass spectrometry as a stability-indicating method for formulation excipient screening for an IgG4 monoclonal antibody. J. Pharm. Sci. 107, 1009–1019. 10.1016/j.xphs.2017.12.009 29269271

[B47] Trabuco L. G. Villa E. Mitra K. Frank J. Schulten K. (2008). Flexible fitting of atomic structures into electron microscopy maps using molecular dynamics. Structure 16, 673–683. 10.1016/j.str.2008.03.005 18462672 PMC2430731

[B48] Trecazzi C. Fisher M. T. (2018). Detecting protein pre-aggregation states using chaperonin biosensor bio-layer interferometry. Am. Pharm. Rev. 21, 52–55.

[B49] Tyagi N. K. Fenton W. A. Deniz A. A. Horwich A. L. (2011). Double mutant MBP refolds at same rate in free solution as inside the GroEL/GroES chaperonin chamber when aggregation in free solution is prevented. FEBS Lett. 585, 1969–1972. 10.1016/j.febslet.2011.05.031 21609718 PMC3144026

[B50] Viitanen P. V. Donaldson G. K. Lorimer G. H. Lubben T. H. Gatenby A. A. (1991). Complex interactions between the chaperonin 60 molecular chaperone and dihydrofolate reductase. Biochemistry 30, 9716–9723. 10.1021/bi00104a021 1680394

[B51] Viitanen P. V. Gatenby A. A. Lorimer G. H. (1992). Purified chaperonin 60 (groEL) interacts with the nonnative states of a multitude of *Escherichia coli* proteins. Protein Sci. 1, 363–369. 10.1002/pro.5560010308 1363913 PMC2142211

[B52] Voziyan P. A. Fisher M. T. (2000). Chaperonin-assisted folding of glutamine synthetase under nonpermissive conditions: off-pathway aggregation propensity does not determine the co-chaperonin requirement. Protein Sci. 9, 2405–2412. 10.1110/ps.9.12.2405 11206062 PMC2144532

[B53] Voziyan P. A. Fisher M. T. (2002). Polyols induce ATP-independent folding of GroEL-bound bacterial glutamine synthetase. Arch. Biochem. Biophys. 397, 293–297. 10.1006/abbi.2001.2620 11795885

[B54] Voziyan P. A. Jadhav L. Fisher M. T. (2000). Refolding a glutamine synthetase truncation mutant in vitro: identifying superior conditions using a combination of chaperonins and osmolytes. J. Pharm. Sci. 89, 1036–1045. 10.1002/1520-6017(200008)89:8<1036::AID-JPS8>3.0.CO;2-5 10906727

[B55] Voziyan P. A. Johnston M. Chao A. Bomhoff G. Fisher M. T. (2005). Designing a high throughput refolding array using a combination of the GroEL chaperonin and osmolytes. J. Struct. Funct. Genomics 6, 183–188. 10.1007/s10969-005-2646-6 16211517

[B56] Wälti M. A. Schmidt T. Murray D. T. Wang H. Hinshaw J. E. Clore G. M. (2017). Chaperonin GroEL accelerates protofibril formation and decorates fibrils of the Het-s prion protein. Proc. Natl. Acad. Sci. U.S.A. 114, 9104–9109. 10.1073/pnas.1711645114 28784759 PMC5576843

[B57] Weaver J. Jiang M. Roth A. Puchalla J. Zhang J. Rye H. S. (2017). GroEL actively stimulates folding of the endogenous substrate protein PepQ. Nat. Commun. 8:15934. 10.1038/ncomms15934 28665408 PMC5497066

[B58] Xia Y. DiPrimio N. Keppel T. R. Vo B. Fraser K. Battaile K. P.. (2013). The designability of protein switches by chemical rescue of structure: mechanisms of inactivation and reactivation. J. Am. Chem. Soc. 135, 18840–18849. 10.1021/ja407644b 24313858 PMC3919134

[B59] Yan X. Maier C. S. (2009). Hydrogen/deuterium exchange mass spectrometry. Methods Mol. Biol. 492, 255–271. 10.1007/978-1-59745-493-3_15 19241038

[B60] Zhang J. H. Chung T. D. Oldenburg K. R. (1999). A simple statistical parameter for use in evaluation and validation of high throughput screening assays. J. Biomol. Screen. 4, 67–73. 10.1177/108705719900400206 10838414

[B61] Zhang Y. (2008). I-TASSER server for protein 3D structure prediction. BMC Bioinformatics 9:40. 10.1186/1471-2105-9-40 18215316 PMC2245901

[B62] Zimmermann M. T. Tischer A. Whitten S. T. Auton M. (2015). Structural origins of misfolding propensity in the platelet adhesive von Willebrand factor A1 domain. Biophys. J. 109, 398–406. 10.1016/j.bpj.2015.06.008 26200876 PMC4621621

[B63] Zolkiewski M. Kessel M. Ginsburg A. Maurizi M. R. (1999). Nucleotide-dependent oligomerization of ClpB from *Escherichia coli*. Protein Sci. 8, 1899–1903. 10.1110/ps.8.9.1899 10493591 PMC2144395

[B64] Zwirowski S. Kłosowska A. Obuchowski I. Nillegoda N. B. Piróg A. Zietkiewicz S.. (2017). Hsp70 displaces small heat shock proteins from aggregates to initiate protein refolding. EMBO J. 36, 783–796. 10.15252/embj.201593378 28219929 PMC5350560

